# Experimental Investigation of Shale Tensile Failure under Thermally Conditioned Linear Fracturing Fluid (LFF) System and Reservoir Temperature Controlled Conditions

**DOI:** 10.3390/polym14122417

**Published:** 2022-06-14

**Authors:** Cajetan Chimezie Iferobia, Maqsood Ahmad, Imtiaz Ali

**Affiliations:** 1 Department of Petroleum Engineering, Universiti Teknologi PETRONAS, Seri Iskandar 32610, Perak Darul Ridzuan, Malaysia; cajetan_17007975@utp.edu.my (C.C.I.); imtiaz_17003333@utp.edu.my (I.A.); 2 Department of Petroleum and Gas Engineering, BUITEMS, Quetta 87300, Balochistan, Pakistan

**Keywords:** linear fracturing fluid, shale gas reservoir, surface crack length, failure pattern, tensile strength

## Abstract

Linear fracturing fluid (LFF) provides viscosity driven benefits of proppant suspensibility and fluid loss control, and with the use of a breaker agent, flowback recovery can be greatly enhanced. Shale tensile strength is critical in the prediction of fracture initiation and propagation, but its behavior under the interaction with LFF at reservoir temperature conditions remains poorly understood. This necessitated an in-depth investigation into the tensile strengths of Eagle Ford and Wolfcamp shales under thermally conditioned LFF and reservoir temperature controlled conditions. Brazilian Indirect Tensile Strength (BITS) testing was carried out for the quantitative evaluation of shale tensile strength, followed by extensive failure pattern classifications and surface crack length analysis. The thermally conditioned LFF saturation of shale samples led to average tensile strength (ATS) increases ranging from 26.33–51.33% for Wolfcamp. Then, for the Eagle Ford samples, ATS increases of 3.94 and 6.79% and decreases of 3.13 and 15.35% were recorded. The exposure of the samples to the temperature condition of 90 °C resulted in ATS increases of 24.46 and 33.78% for Eagle Ford and Wolfcamp shales, respectively. Then, for samples exposed to 220 °C, ATS decreases of 6.11 and 5.32% were respectively recorded for Eagle Ford and Wolfcamp shales. The experimental results of this research will facilitate models’ development towards tensile strength predictions and failure pattern analysis and quantifications in the LFF driven hydraulic fracturing of shale gas reservoirs.

## 1. Introduction

Water-based fracturing fluids have been the main drivers of shale gas reservoirs’ hydraulic fracturing, with slickwater being more widely used in comparison to linear fracturing fluid of remarkable viscosity. Slickwater utilization leads to the generation of highly conductive fractures [1], easy flowback, highly stimulated reservoir volume [2], and effective cost savings [3]. However, it requires large water volumes and is equally associated with poor proppant carrying capacity, the creation of narrow fracture widths, and high leak-off due to minimal wall building [4]. Linear fracturing fluid with the benefit of higher viscosity ensures proppant suspensibility, better fluid loss control, and maximized flowback recovery achieved by breaker agent utilization. 

Linear fracturing fluid (LFF) is formulated by the addition of a polymeric agent into an aqueous solution for viscous gel formation. Other chemical additives could be introduced for the enhancement of fluid properties. Guar gum and its derivatives are widely used polymers in the industry [5] for hydraulic fracturing fluid preparations. However, certain draw backs are encountered, such as the high cost and significant amounts of polymeric residues being left after fluid breakdown [6]. These polymeric residues have tendencies to cause damages to the proppant pack and equally result in the poor conductivity of fracture networks [7,8,9].

The productivity of shale gas reservoirs remains highly dependent on the efficient design of hydraulic fracturing treatment, which requires a good understanding of shale geomechanical properties [10]. Tensile strength is a critical geomechanical property that has significant control in the opening and propagation of hydraulic fractures [11,12]. It was originally quantified using a direct uniaxial tensile strength test, which requires a special type of rock specimen [13]. The challenges associated with the construction of this specimen led to the development and application of Brazilian Indirect Tensile Strength (BITS) testing for the tensile strength evaluation of rock materials [14]. BITS testing requires the application of linearly concentrated compressive loads across the diameter of a disc-shaped sample to failure, with the length-to-diameter ratio of the sample being in the range of 0.20–0.75 [15]. 

Several indirect methods of tensile strength determination have been developed, such as the ring test, hoop test, bending test, and hydraulic extension test [16]. However, BITS testing, recognized worldwide, has remained the key standard and is the preferred indirect test method for the determination of rock tensile strength. During BITS testing, fractures and cracks are initiated at the loading points of a specimen, and these fractures and cracks propagate along the loaded diameter of the specimen to complete failure. 

Researchers have investigated (1) burial depth [17,18], (2) total organic carbon (TOC) [19,20], (3) bedding and/or loading orientations [12,20,21,22,23,24,25,26], and (4) KCl-water saturation [12] effects on shale tensile strength. These established that studies relating to shale tensile failure have focused mainly on bedding and/or loading orientation effects on shale tensile strength. This heightened the need to provide a comprehensive understanding on the responses of shale tensile strength to thermally conditioned linear fracturing fluid and reservoir temperature controlled conditions. 

In this research, linear fracturing fluid was formulated using sodium carboxyl methyl cellulose (Na-CMC) as a polymeric agent, due to its minimal residue production in comparison to guar gum. Potassium chloride (KCl) acted as a clay stabilizer, and hydrolyzed polyacrylamide (HPAM) functioned as a friction reducer. Shale samples were obtained from USA’s Eagle Ford and Wolfcamp shales, known for their commercial gas reserves. The mineralogical characterization, elemental composition analysis, and porosity–permeability evaluation of samples were carried out. BITS testing was conducted for the tensile strength evaluation of shale samples after subjection to various treatment conditions. An in-depth failure analysis and classifications, along with surface crack length measurements, were conducted. This research will provide significant levels of datasets to drive models’ development towards tensile strength predictions and failure pattern analysis and quantifications in LFF driven hydraulic fracturing of shale gas reservoirs. 

## 2. Statement of Theories

Shale has been taken to be anisotropic [27], and efforts have been made by several researchers to account for the tensile strength of anisotropic (transversely isotropic) rocks. Amadei and Jonsson [28] and Chen et al. [29] utilized the complex stress function method of Lekhnitskii et al. [30] to account for the tensile strength of anisotropic rocks under diametrical compression. Chen et al. [29] proposed an analytical solution which required the use of stress charts to account for anisotropic rock tensile strength. Duan and Kwok [11] described the analytical solution of Chen et al. [29] as implicit and required a numerical method to account for the stress field. Claesson and Bohloli [31] improved the analytical solution of Chen et al. [29] and derived an approximate expression for the principal stresses acting at the disc center. Lee and Pietruszczak [32] and Ma et al. [13] reported that these analytical solutions for anisotropic rock tensile strength determination remain less developed due to the difficulty of experimental validations. 

BITS testing has been used in the quantitative evaluation of shale tensile strength under different conditions [24,33,34,35,36]. BITS is determined based on the assumption of the isotropy, homogeneity, and linear elasticity of rock materials [11,14]. BITS may not fully represent the tensile strength of shale given its anisotropic properties. However, BITS testing was applied in this case to aid the comparability of shales’ tensile strengths, following exposure to thermally conditioned LFF and reservoir temperature controlled conditions. 

**Figure 1:**
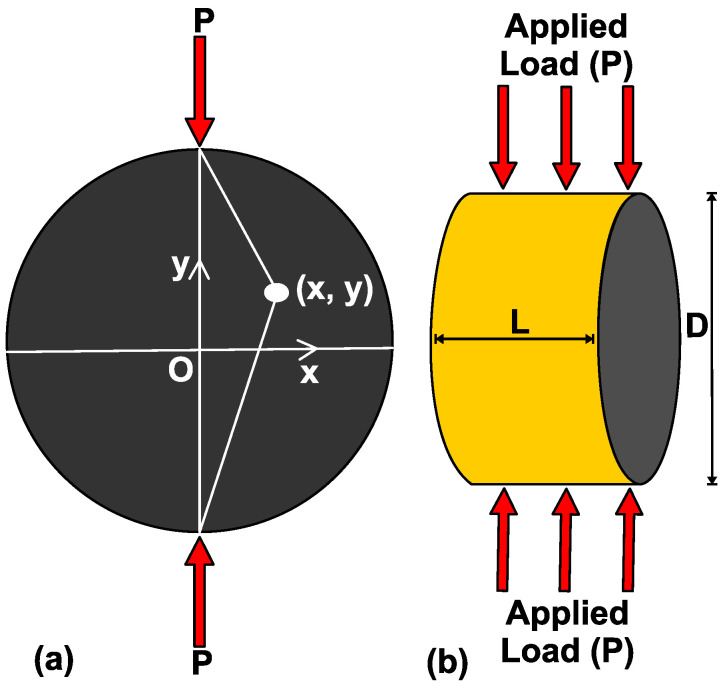
BITS testing’s associated parametric configurations. (**a**) State of stress at point (x, y) on an isotropic Brazilian disc, and (**b**) diametrical compressional loading of the test specimen with diameter (D) and thickness (L) (Modified after: Li et al. [12]).

The tensile strengths of anisotropic rocks have been investigated based on BITS testing [24,31,33,34,35,36]. BITS can be determined using Equation (1), as suggested by ASTM [15] and ISRM [37] and in adherence to the theory of isotropic elasticity. In Equation (1), *P* in Newton (N) represented the specimen applied failure load, *D* represented the diameter of the test specimen in mm, *L* represented the thickness of the specimen in mm, and $\sigma_{t}$ represented the tensile strength in MPa or N/mm^2^. $\sigma_{t}$ can be converted to psi by the multiplication of Equation (1) by a constant value (145.038), as represented in Equation (2) [12,26]. Li et al. [12] reported on the analytical solution of a stress state at the point (x, y) on an isotropic Brazilian disc (Figure 1a) under diametrical compressional loading driven by the top-bottom platens (Figure 1b).

(1) $$\sigma_{t}=\frac{2P}{\pi DL}$$

(2) $$\sigma_{t}=145.038*\frac{2P}{\pi DL}$$

## 3. Methodology

### 3.1. Geological Setting

**Figure 2:**
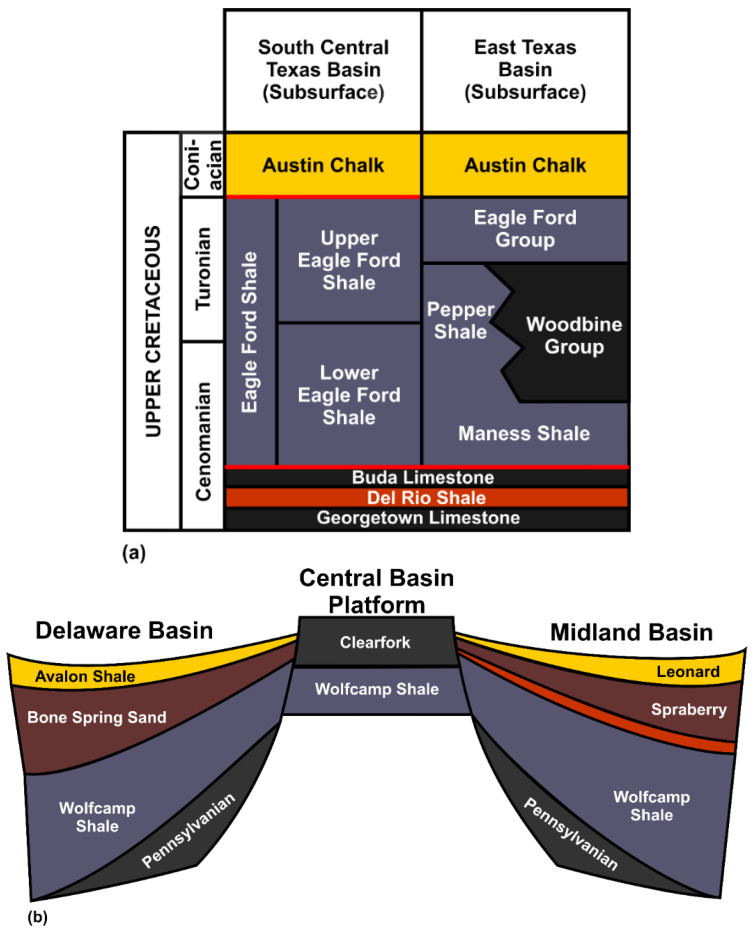
(**a**) Stratigraphy of the Eagle Ford Shale (Modified after: Workman [42]), and (**b**) Stratigraphy of the Wolfcamp shale from the Delaware Basin, extending through the Central Basin Platform to the Midland Basin (Modified after: Sutton [41]).

The shale samples used in this research were outcrop samples of USA’s Eagle Ford and Wolfcamp shale formations, known for their commercial shale gas reserves. Eagle Ford was discovered as a late cretaceous organic rich shale. It underlies much of South Texas and extends about 400 miles from the Texas–Mexico border in the Webb and Maverick Counties to East Texas [38]. The two major depositional units of the Eagle Ford are (1) oil prone transgressive, and (2) gas prone regressive units. These units were described as the Lower and Upper Eagle Fords, respectively [39], and the stratigraphy of the Eagle Ford shale is contained in Figure 2a. Wolfcamp shale is situated in the Permian Basin, which is rated as one of the biggest basins in the world. Permian Basin is associated with two large sub-basins (Delaware and Midland Basins) separated by the Central Basin Platform [40,41]. The vertical plugs (cored perpendicular to bedding) from the Eagle Ford and Wolfcamp shales (Figure S1 in the Supplementary Materials) were used in the construction of the Brazilian disc-shaped samples utilized in this research. 

### 3.2. Samples Characterization

**Figure 3:**
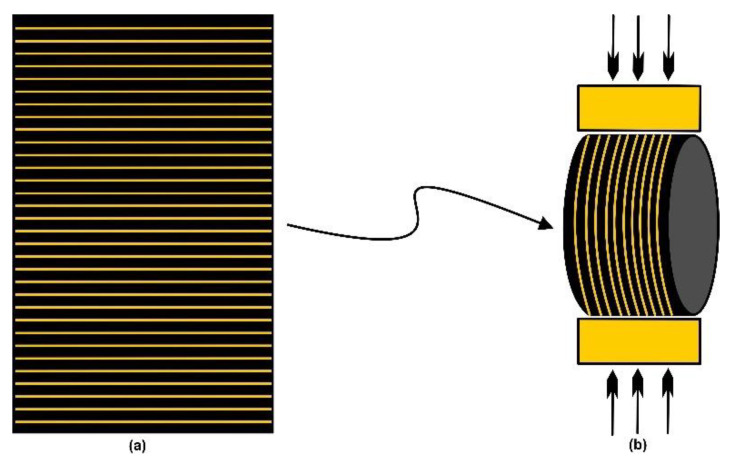
Schematic representations of (**a**) a vertical plug, and (**b**) a Brazilian disc-shaped sample.

A total of 32 Brazilian disc-shaped samples were used in this research and in strict adherence to a length-to-diameter ratio within 0.20–0.75, in compliance with ASTM standards [15]. The Brazilian disc-shaped samples are comprised of 16 samples from the Eagle Ford shale and 16 samples from the Wolfcamp shale. These samples were cut from cylindrically shaped plugs of perpendicular orientations to bedding. Figure 3a is the schematic representation of a vertical plug used in the construction of a Brazilian disc-shaped sample (Figure 3b), with compressional loading across its bedding orientation. 

A field emission scanning electron microscope (FESEM), Supra 55VP model, was used in the examination of shale samples’ mineralogical structures. Elemental analysis, mapping and visualization were accomplished using an external Energy Dispersive X-ray spectrometer (EDX). The splits of the shale samples of weight (<1.0 g each) were individually coated with gold for the provision of conductive layering using a K550X sputter coater model. The imaging and analysis of samples were carried out using a FESEM-EDX integrated system. Mercury Intrusion Porosimetry (MIP) was conducted for the porosity-permeability characterization of samples using Pascal 140 and 240 Series Mercury Porosimeters. The set-up of the FESEM-EDX, Pascal 140 and 240 Series Mercury Porosimeters, and sputter coater is contained in the Supplementary Materials (Figure S2). 

### 3.3. Fluid Preparation and Shale Samples’ Treatment

Linear fracturing fluids (LFFs) were formulated using two different concentrations (0.65 and 2.50%) of polymeric agent to account for low and high viscosity linear fracturing fluids, respectively. In the formulation of LFF, HPAM at 0.2% functioned as a friction reducer, KCl at 2% was used for clay stabilization [43], and water served as the base fluid. Industrial grade Na-CMC served as the polymeric agent. The complete dissolution of chemical additives (Na-CMC, HPAM, and KCl) was achieved with the use of a Model 9B 5-spindle Multimixer. Brazilian disc-shaped shale samples were positioned uprightly in pairs inside borosilicate glass containers before the introduction of linear fracturing fluids. Afterwards, the containers were covered with aluminum foil and tightened with metallic lids. 

The containers were then placed in a Binder Heating Oven system at a temperature of 90 °C for periods of 5 and 20 days. These periods represented short and long (in case of down time) term periods of hydraulic fracturing, respectively. The Brazilian disc-shaped samples were equally exposed to controlled reservoir temperature conditions of 90 and 220 °C using the Binder Heating Oven system and CWF model Chamber Furnace, respectively. Shale samples at the completion of thermally conditioned linear fracturing fluid saturations and exposure to reservoir temperature controlled conditions were subjected to BITS testing for the evaluation of tensile strengths. The set-up of the Model 9B 5-Spindle Multimixer, Binder oven, and Carbolite chamber furnace is contained in the Supplementary Materials (Figure S3).

**Figure 4:**
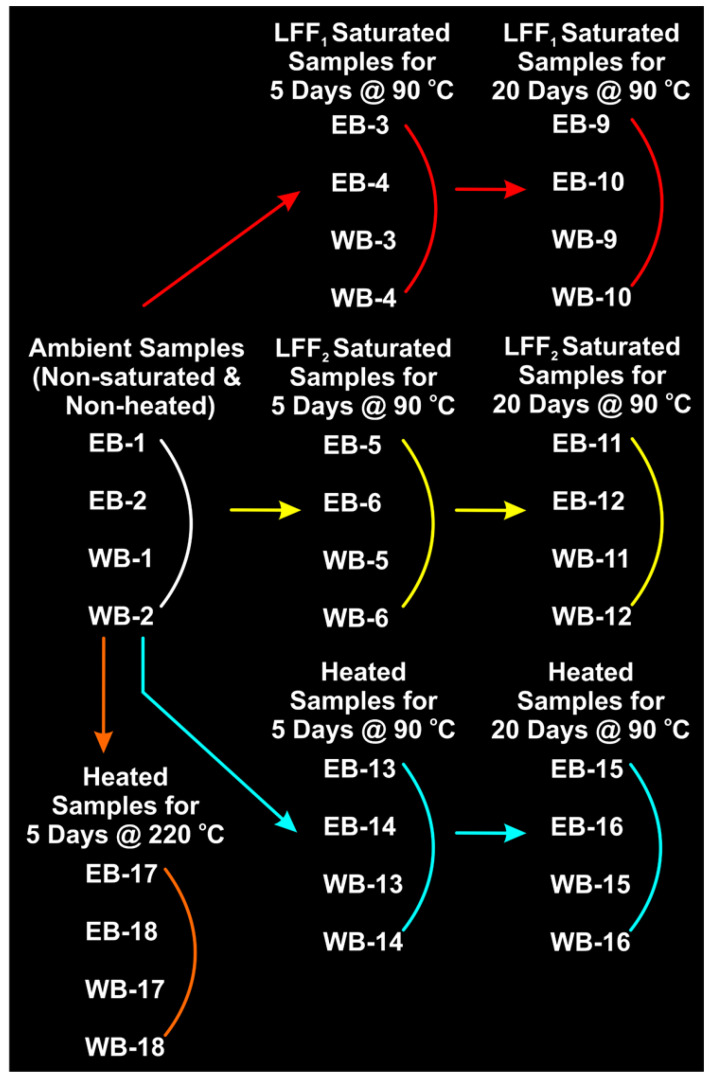
Brazilian disc-shaped samples of Eagle Ford (EB) and Wolfcamp (WB) shales with indications of associated treatment conditions. Samples EB-7, EB-8, WB-7, and WB-8 were damaged upon handling and were excluded from this research.

Figure S4 in the Supplementary Materials represented the Brazilian disc-shaped samples of Eagle Ford and Wolfcamp shales exposed to linear fracturing fluids at polymer concentrations of 0.65 and 2.50%. The black markings on the samples defined centerlines and compressional load directions. Figure 4 is a representation of the various samples and associated treatment conditions before BITS testing. The samples of Eagle Ford were designated as EB and those of Wolfcamp as WB. LFF_1_ and LFF_2_ represented linear fracturing fluids at polymer concentrations of 0.65 and 2.50%, respectively. Ambient or reference samples referred to zero fluid saturated and zero heat treated samples. 

### 3.4. Brazilian Indirect Tensile Strength Testing 

Unitronic Compression–Tensile Equipment (S205 Model) with a rated maximum load of 25 kilonewton (kN) was used in the BITS testing of the shale samples. It has a frontally placed control panel fitted with six multifunctional interactive pushbuttons and a large graphic display. The Brazilian disc-shaped sample is accommodated in a sample holder and is loaded to failure with on-the-spot display of the deformation and maximum load for tensile strength evaluation. The equipment set-up can be found in the Supplementary Materials (Figure S5). 

## 4. Results Analysis and Discussion

### 4.1. FESEM-EDX-Mapping Analysis of Eagle Ford and Wolfcamp Shales

**Figure 5:**
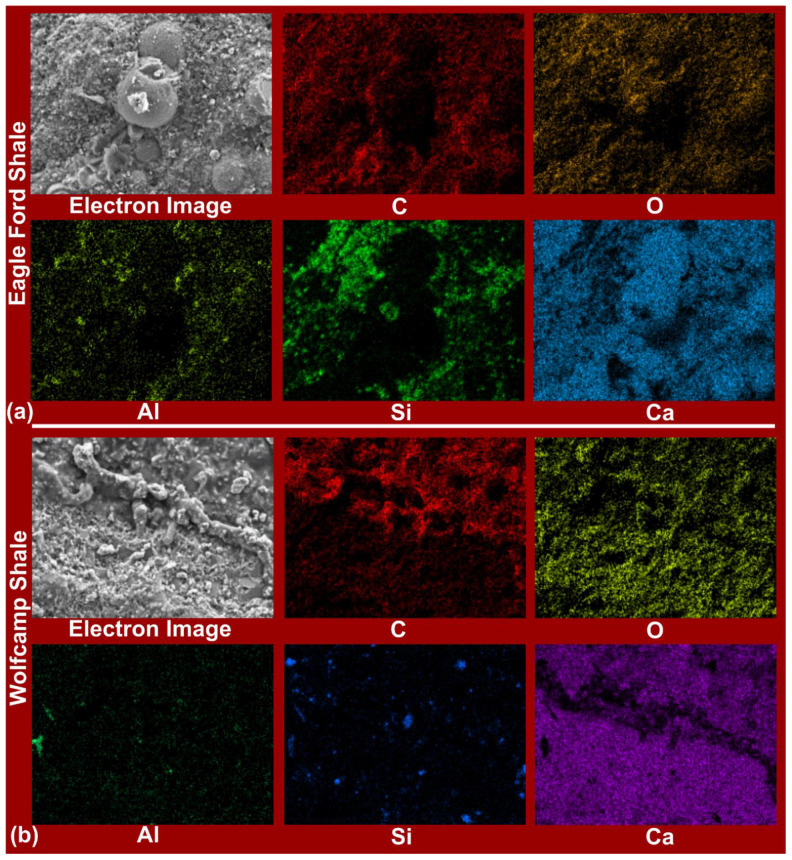
Elemental distribution maps of (**a**) Eagle Ford shale and (**b**) Wolfcamp shale. Detected elements were represented with their chemical symbols (C: carbon, O: oxygen, Al: aluminum, Si: silicon, and Ca: calcite).

FESEM-EDX-Mapping facilitated the mineralogical and elemental distribution analyses of Eagle Ford and Wolfcamp shales. In Figure 5a, an elemental map of the Eagle Ford shale revealed the distributions of C, O, Al, Si, and Ca. Ca, C, and O exhibited dominance and suggested the predominant presence of calcite (CaCO_3_). The Si and O distributions supported the presence of quartz (SiO_2_). The less dense distribution of Al in combination with Si and O suggested the likely presence of kaolinite (Al_2_Si_2_O_5_(OH)_4_), but under the consideration of Ca, supported the possible presence of plagioclase (CaAl_2_Si_2_O_8_). Ali and Hascakir [44], Ramiro-Ramirez [39], and Workman [42] reported the presence of these minerals in Eagle Ford shale. 

Based on the elemental map of Wolfcamp shale as contained in Figure 5b, the dominant distributions of Ca, C, and O supported the significant presence of calcite, and Jones [40] reported on the domineering presence of calcite in Wolfcamp shale. Si and Al were more densely distributed in Eagle Ford (Figure 5a) than in Wolfcamp shale (Figure 5b), suggesting the richer presence of quartz and kaolinite and/or plagioclase in the Eagle Ford shale. Owing to the rich distributions of the elements Ca, C, and O, in Figure 5a,b, it can be seen that calcite is richly present in the Eagle Ford and Wolfcamp shales. 

**Figure 6:**
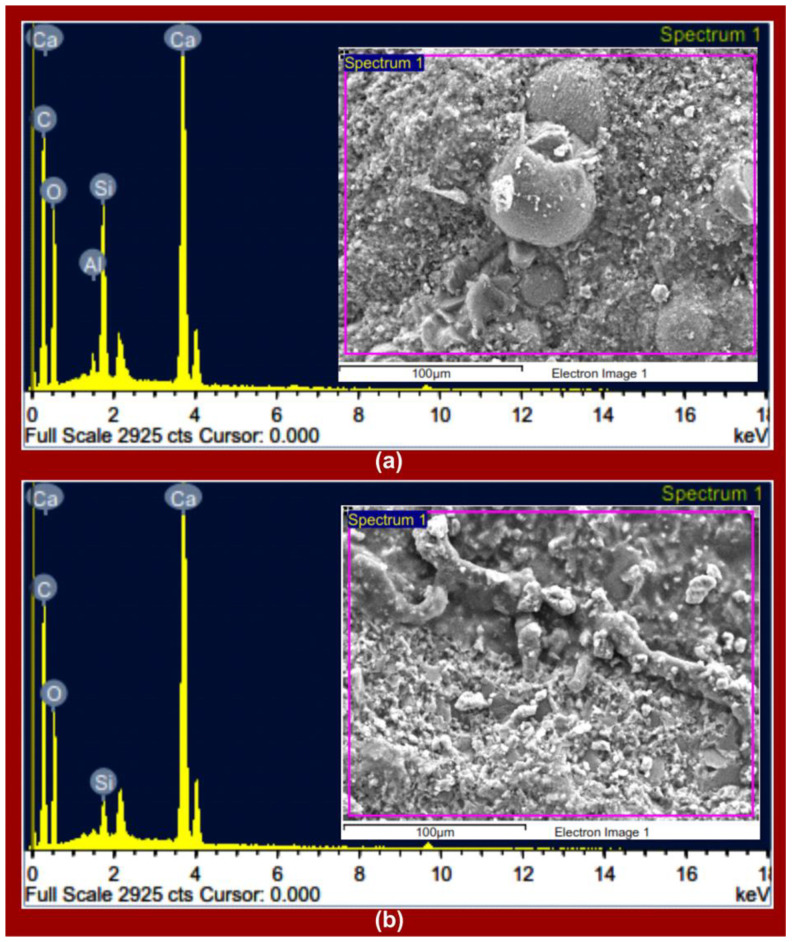
EDX analyses of (**a**) Eagle Ford shale and (**b**) Wolfcamp shale, with indications of peak intensities associated with detected elements.

**Figure 7:**
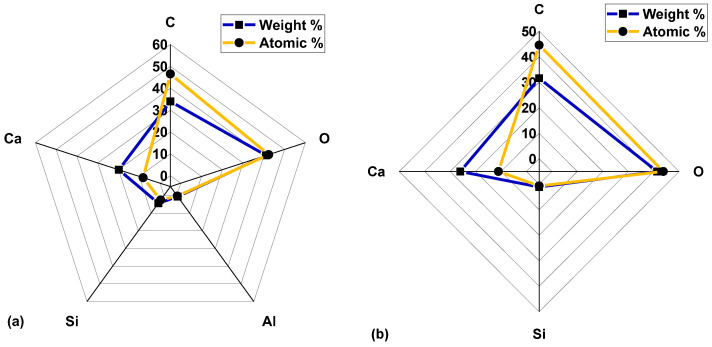
(**a**) Eagle Ford and (**b**) Wolfcamp shales’ detected elements and their associated weights and atomic percentages.

EDX analyses of the Eagle Ford and Wolfcamp shales (Figure 6a,b), respectively, demonstrated the dominance of Ca, C, and O, as indicated by their associated peak intensities. This validated the elemental mapping analyses contained in Figure 5. The weight percentage (wt.%) and atomic percentage (at. %) of Ca, C, and O amounted to 94.91 and 97%, respectively, in the Eagle Ford shale (Figure 7a). For the Wolfcamp shale, they amounted to 98.88 wt. % and 99.33 at. % (Figure 7b). This suggested greater calcite dominance in the Wolfcamp shale than in the Eagle Ford shale. Si accounted for 4.41 wt. % and 2.59 at. % in the Eagle Ford shale (Figure 7a) and 1.12 wt. % and 0.67 at. % in the Wolfcamp shale (Figure 7b). This supported the likely tendencies of higher quartz content in the Eagle Ford shale. 

**Figure 8:**
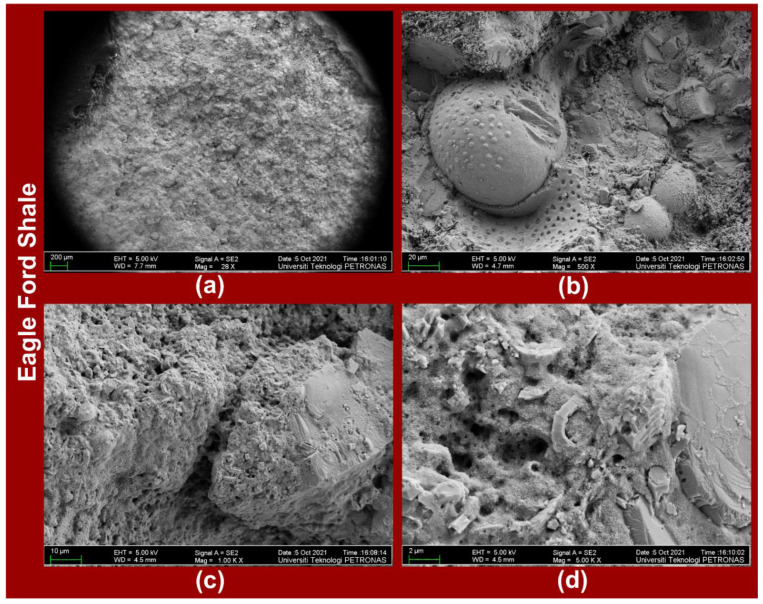
FESEM images of Eagle Ford shale at magnifications of (**a**) 28X, (**b**) 500X, (**c**) 1000X, and (**d**) 5000X.

**Figure 9:**
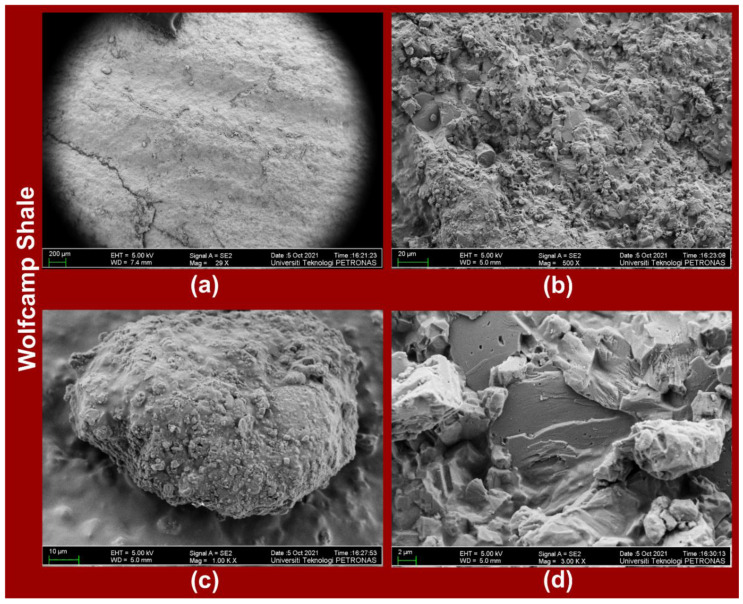
FESEM images of Wolfcamp shale at magnifications of (**a**) 29X, (**b**) 500X, (**c**) 1000X, and (**d**) 3000X.

The FESEM images of the Eagle Ford shale (Figure 8a–d) at respective magnifications of 28X, 500X, 1000X, and 5000X and those of the Wolfcamp shale (Figure 9a–d) at respective magnifications of 29X, 500X, 1000X, and 3000X displayed the variabilities in the shales’ mineralogical structures. The variability in the mineralogical structures is more evident in the Eagle Ford shale (Figure 8b,d) than in the Wolfcamp shale (Figure 9b,d). The Wolfcamp shale revealed the presence of natural fractures in Figure 9a at a magnification of 29X. 

### 4.2. Porosity—Permeability Characterization of Eagle Ford and Wolfcamp Shales

Pascal 140 and 240 Series Mercury Porosimeters were used for mercury intrusion into the pore networks of Eagle Ford and Wolfcamp shale samples. This was to evaluate porosity, its related parameters (pore diameter, volume, and surface area distributions), and permeability. Anovitz and Cole [45] reported on the classifications of pore diameters into micropores (<2 nm), mesopores (2–50 nm), and macropores (>50 nm) to guide pore size distribution measurements. Experimental data resulting from MIP are graphically represented using curves of cumulative, incremental, and/or differential pore volume distributions with respect to pore size [46]. Based on these graphical representations, pore size range, peak pore size distribution, and dominant pore size are quantitatively evaluated. 

**Figure 10:**
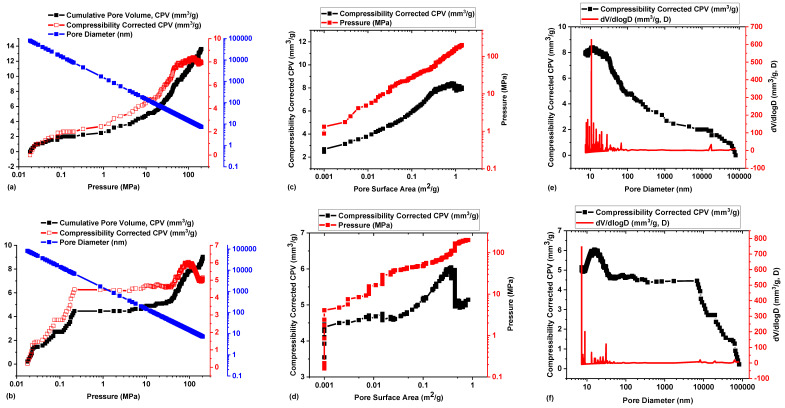
Pore system characterization of shale samples. (**a**) Eagle Ford and (**b**) Wolfcamp shales’ CPV and CCCPV versus mercury intrusion pressure, (**c**) Eagle Ford and (**d**) Wolfcamp shales’ CCCPV and mercury intrusion pressure versus PSA, and (**e**) Eagle Ford and (**f**) Wolfcamp shales’ CCCPV and dV/dlogD versus pore diameter.

Eagle Ford shale had a cumulative pore volume (CPV) and compressibility corrected CPV (CCCPV) of 13.62 and 7.90 mm^3^/g, respectively, at a mercury intrusion pressure of 202.4174 MPa (Figure 10a). The pore diameter sizes following the intrusion ranged from 7.27–79,978.54 nm for the Eagle Ford shale (Figure 10a). Pores of smaller diameters were densely concentrated at regions of high mercury intrusion pressures, while those of large diameter sizes were concentrated at the regions of low mercury intrusion pressures (Figure 10a). In Figure 10b, Wolfcamp shale had CPV and CCCPV values of 9.03 and 5.14 mm^3^/g, respectively, at an intrusion pressure of 202.2174 MPa, which implied that the pore volume distribution of the Eagle Ford shale was higher than that of the Wolfcamp shale. 

The pore diameters associated with the Wolfcamp shale ranged from 7.27–82,490.48 nm (Figure 10b), with a high concentration of small diameter pores at high intrusion pressures and a high concentration of large diameter pores at low pressures. The pore surface areas (PSAs) of the shale samples (Eagle Ford and Wolfcamp) were more exposed at high intrusion pressures. Eagle Ford had a PSA value of (1.39 m^2^/g) at its maximum intrusion pressure (Figure 10c), while Wolfcamp shale was of lower PSA (0.816 m^2^/g) at its maximum intrusion pressure (Figure 10d). 

In Figure 10e,f, the respective plots of CCCPV versus pore diameter (PD) and log differential pore volume distribution (dV/dlogD) versus PD demonstrated the dominance of small pore diameter sizes over large diameter pores. These plots provided an avenue for the determination of average, median, and modal pore diameters. The Eagle Ford shale recorded an average pore diameter (APD) of 22.74 nm, a median pore diameter of 258.36 nm, and a modal pore diameter of 10.64 nm (Figure 10e). Wolfcamp shale had 25.22 nm for an average pore diameter, 20,164.11 nm for a median pore diameter, and 7.27 nm for a modal pore diameter (Figure 10f). The average pore diameters for the Eagle Ford and Wolfcamp shales were thus classified to be in the mesopore region. 

Based on the MIP experiments, the Eagle Ford and Wolfcamp shales’ accessible porosities were determined. Eagle Ford shale recorded an accessible porosity of 2.95% without compressibility correction, and, upon compressibility correction, it became 1.71%. Wolfcamp shale’s accessible porosity without compressibility correction was 1.61% and after correction became 0.91%. General permeabilities were equally evaluated for the Eagle Ford and Wolfcamp shales under the consideration of tortuosity factor. Eagle Ford shale had a general permeability of 2.50 × 10^−5^ µm^2^, while that of Wolfcamp was 1.64 × 10^−5^ µm^2^. Zou et al. [47] reported the porosity and permeability being at <6% and <0.5 × 10^−3^ µm^2^, respectively, for shale gas reservoirs; thus, the reported values are in line. 

MIP experimental data analysis of the Eagle Ford and Wolfcamp shales has thus established that the Eagle Ford shale was of a higher pore volume and pore surface area distributions and was associated with higher porosity and permeability in comparison to the Wolfcamp shale. MIP experimentation has proven to be a reliable and competent approach for the in-depth analysis of pore size distributions and porosity–permeability characterizations of Eagle Ford and Wolfcamp shales. 

### 4.3. Thermal Conditioning Effects on Physical Appearances of Shale and LFF

**Figure 11:**
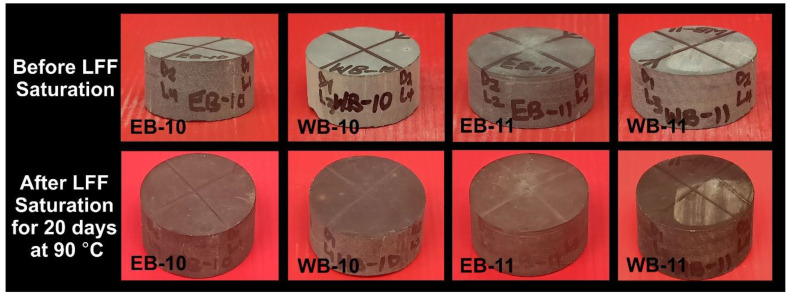
Pre- and Post-LFF saturations of Eagle Ford and Wolfcamp shales at 90 °C for a period of 20 days. EB-10 and WB-10 were LFF_1_ saturated, while EB-11 and WB-11 were LFF_2_ saturated.

**Figure 12:**
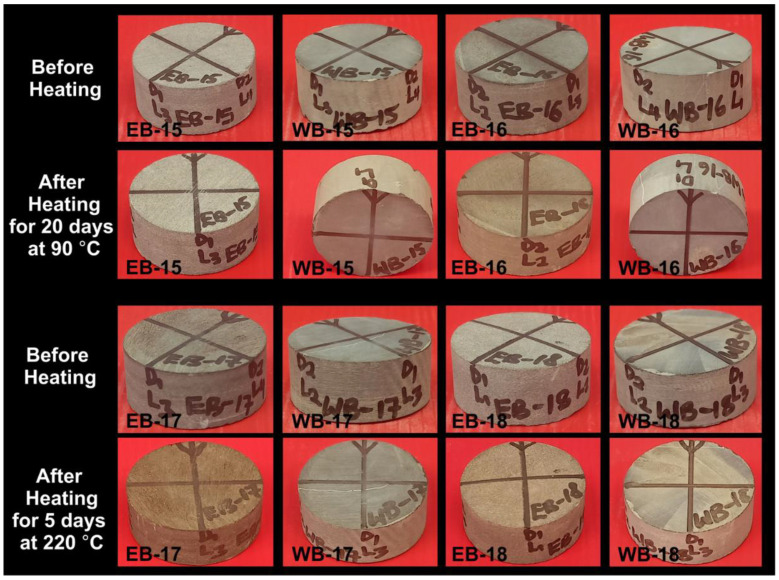
Pre- and Post-thermal conditioning of Eagle Ford and Wolfcamp shales for the periods of 20 days at 90 °C for EB-15, EB-16, WB-15, and WB-16 and 5 days at 220 °C for EB-17, EB-18, WB-17, and WB-18.

A total of 12 samples were selected for physical examinations (Figure 11, Figure 12). Samples EB-10 and WB-10 were LFF_1_ saturated, and EB-11 and WB-11 were LFF_2_ saturated, all for 20 days at a temperature of 90 °C (Figure 11). Samples’ coloration changes from light to deep grey are evident in Figure 11. These changes in coloration can be attributed to samples absorption of LFF, as indicated by the increase in the masses of the samples at the end of saturation period. Samples EB-15, EB-16, WB-15, and WB-16 were heated for 20 days at 90 °C, and EB-17, EB-18, WB-17, and WB-18 were heated for 5 days at 220 °C (Figure 12). The heating of these samples resulted in coloration changes that were very visible on samples EB-16, EB-17, EB-18, and WB-18 (Figure 12). 

The losses in mass resulting from the heating of these samples demonstrated true evidence of pore fluid dehydration, triggering mineralogical alteration, and inducing samples’ coloration changes. Kang et al. [48] reported that changes in mass after the heat treatment of rock samples are true reflections of samples’ free water, adsorbed water, interlayer water, and/or constitution water losses. Idris [49] and Saiang and Miskovsky [50] reported on the evaporation of free water in the pre-existing pores of rock samples on exposure to thermal conditions over a period of time. Liu and Xu [51] reported on rock color changes owing to exposure to thermal conditions.

**Figure 13:**
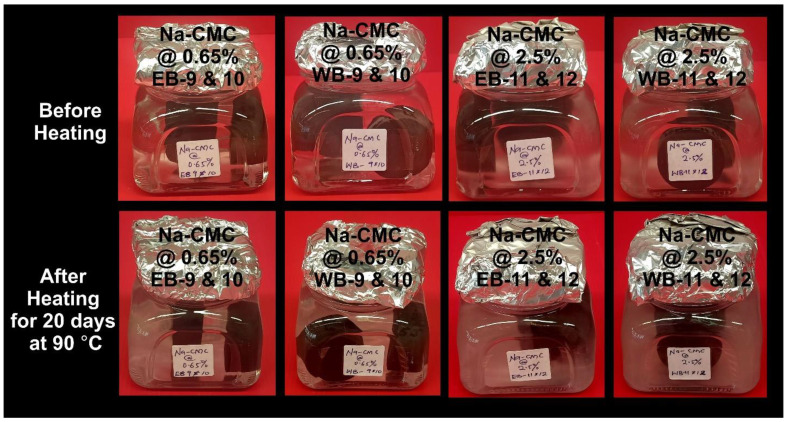
Borosilicate glass containers housing paired samples exposed to LFF at 90 °C for a period of 20 days.

Borosilicate glass containers were used in housing the samples in pairs (EB-9 & 10, WB-9 & 10, EB-11 & 12, and WB-11 & 12) during LFF saturation at 90 °C for 20 days (Figure 13). The samples were exposed to LFF of different polymeric concentrations (LFF_1_ at 0.65% Na-CMC and LFF_2_ at 2.50% Na-CMC). There were no highly visible changes in LFF_1_ after exposure to thermal conditions, but LFF_2_ showed a color change from near-colorless to light white after thermal exposure. This implied that LFF_2_, due to the higher polymeric concentration, experienced more reactivity than the LFF_1_ of lower Na-CMC concentration. 

### 4.4. Tensile Strength and Failure Analysis of Eagle Ford and Wolfcamp Shales

#### 4.4.1. Tensile Strength and Failure Analysis of Thermally Conditioned LFF_1_ Saturated Samples

**Figure 14:**
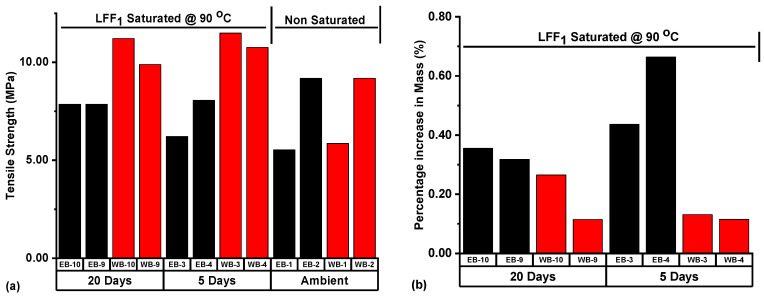

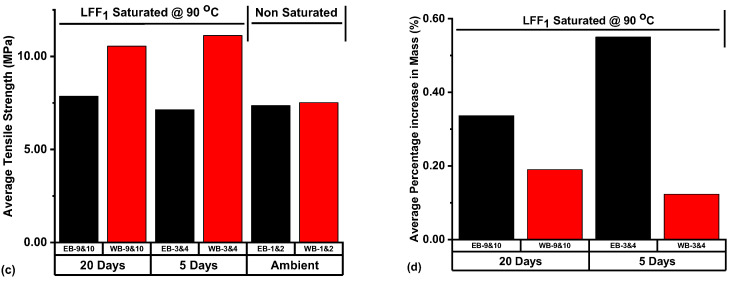
(**a**) Tensile strength, (**b**) Percentage increase in mass, (**c**) Average tensile strength (ATS), and (**d**) Average percentage increase in mass (APIM) of samples subjected to different treatment conditions prior to BITS testing. Notes: (1) Ambient samples were non-saturated and non-heated, and (2) All reported values were corrected to 2 decimal places after calculations, and, due to this, a reported average value may vary by 0.01 from the average value to be obtained using values already corrected to 2 decimal places.

In Figure 14a, the Wolfcamp sample (WB-3) under 5 days of LFF_1_ saturation at 90 °C had the highest tensile strength (11.49 MPa), followed by WB-10 under 20 days of LFF_1_ saturation at 90 °C, with a tensile strength of 11.21 MPa. In Figure 14b, EB-4 under 5 days of LFF_1_ saturation at 90 °C had the highest percentage increase in mass at 0.66%, followed by EB-3 at 0.44%, while the Wolfcamp sample (WB-9) under 20 days of LFF_1_ saturation at 90 °C had the lowest (0.11%). Based on Figure 14a,b, the Wolfcamp samples in comparison to the Eagle Ford samples are more associated with higher values of tensile strength and lower values of percentage increase in mass. 

Figure 14c,d showcased the respective average tensile strength (ATS) and average percentage increase in mass (APIM) resulting from the Eagle Ford and Wolfcamp samples being subjected to different treatment conditions prior to BITS testing. The ATS of the ambient Wolfcamp samples (WB-1 & 2) was 7.52 MPa, and the ATS of WB-3 & 4, with an APIM at 0.12% under 5 days of LFF_1_ saturation at 90 °C, was 11.13 MPa. This represented an ATS increase of 48.01%. Then, under 20 days of LFF_1_ saturation at 90 °C, the ATS of WB-9 & 10, with an APIM at 0.19%, was 10.55 MPa. This represented an ATS increase of 40.29% with respect to the ATS of ambient samples WB-1 & 2. LFF_1_ saturation at 90 °C led to an increase in the ATS of the Wolfcamp samples by 48.01% for WB-3 & 4 and by 40.29% for WB-9 & 10, with reference to the ATS of ambient samples WB-1 & 2. Wolfcamp samples (WB-3 & 4) with lower APIM (0.12%) were associated with higher ATS (11.13 MPa), while WB-9 & 10, with higher APIM (0.19%), had lower ATS (10.55 MPa). This indicated that the higher absorption of LFF_1_ at 90 °C, as reflected by the APIM of WB-9 & 10, led to gradual decrease in the ATS in WB-9 & 10, with reference to the ATS of WB-3 & 4. 

In the case of ambient Eagle Ford samples EB-1 & 2, the ATS was at 7.36 MPa. EB-3 & 4 under 5 days of LFF_1_ saturation at 90 °C had an APIM of 0.55% and an ATS value of 7.13 MPa. This represented an ATS decrease of 3.13%, with reference to the ATS of EB-1 & 2. Eagle Ford samples EB-9 & 10, with an APIM of 0.34% under 20 days of LFF_1_ saturation at 90 °C, had an ATS value of 7.86 MPa, an increase of 6.79% in ATS with respect to the ATS of ambient samples EB-1 & 2. It can be seen that the ATS of the Eagle Ford samples was higher at lower APIM and lower at higher APIM (Figure 14c,d). This was equally demonstrated by the Wolfcamp samples WB-3 & 4, which had a higher ATS (11.13 MPa) at a lower APIM (0.12%) (Figure 14c,d). It was expected that the APIM values would be highest during 20 days of LFF_1_ saturation at 90 °C, but from Figure 14d, the APIM of EB-3 & 4 under 5 days of LFF_1_ saturation was very high at 0.55%, and it became 0.34% for EB-9 & 10 under 20 days saturation. This can be attributed to a possible higher degree of accessible pores present in EB-3 & 4 than in samples EB-9 & 10, thus encouraged larger quantities of fluid entrapment.

LFF_1_, due to its adhesiveness, viscoelasticity, and gel strength characteristics induced by the Na-CMC polymeric agent, is liable to cause the compaction of shale grains to boost the tensile strength of samples. Bailey et al. [52], Grillet et al. [53], Karakul [54], Kropka et al. [55], and Zosel [56] reported that polymer develops adhesion upon contact with a surface. This adhesion will necessitate the strengthening of material properties when the rock is under interaction with polymer containing fluid [54,56]. This is liable for the ATS increases resulting from the LFF_1_ saturation of Eagle Ford and Wolfcamp shales at 90 °C. Al-Shajalee et al. [57] and Mishra et al. [58] equally reported that the increase of pore fluid saturation levels has the capacity to decrease the adhesion potentials of polymer containing fluid upon interaction with a rock material surface. This could possibly be liable for the gradual reduction in samples’ ATS at higher levels of APIM, as witnessed in Figure 14c,d. 

Different failure patterns have been attributed to diameter-concentrated compressional loading to the failure of Brazilian disc-shaped samples, which are either isotropic or anisotropic in nature. Li et al. [12] reported on linearly shaped primary fractures, which occur along or near the centerline of rock samples. The resulting fracture planes are parallel and perpendicular to the directions of compressional load and tensile stresses, respectively. Li et al. [12] equally reported on the likely occurrences of secondary fractures along laminations and shear failures driven by edge-concentrated compressional loads. Hou et al. [23] and Ma et al. [13] reported on tensile splitting, tensile-shear, and shear failure patterns. Tensile splitting is synonymous with primary fractures, and tensile-shear failure with a half-moon shaped fracture plane may be caused by the suppression of rock failure due to the high bonding strength between the rock’s bedding layers. Shear failure is associated with a short curve-shaped fracture plane due to fracture surface slipping along bedding planes with shearing playing a leading role in the rock’s failure process. 

Basu et al. [59] and Tavallali and Vervoort [60] reported on three (3) failure patterns (layer activation, central fracture, and non-central fracture) associated with disc specimens under Brazilian tensile testing conditions. Layer activation results in fractures that are parallel to the foliation plane since foliations provide easily accessible sites for the faster release of stored strain energy. Central fractures are centralized and parallel to loading directions, while non-central fractures are non-centralized curve/arc shaped fractures, starting at or around loading platens. They represent strong indications of high tensile strength tendencies and occur due to the challenging exploitation of rock laminations for the release of stored strain energy. 

**Figure 15:**
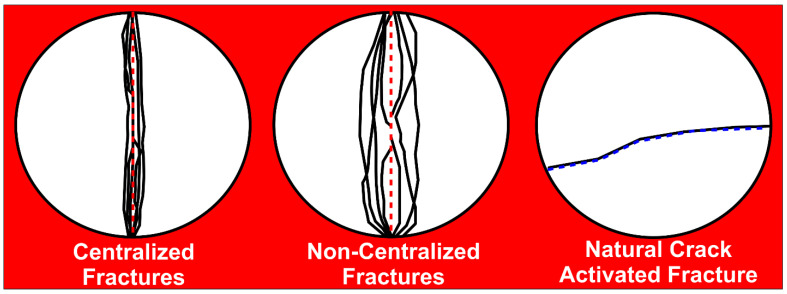
Schematic representation of failure patterns arising from the Brazilian tensile testing of disc-shaped samples with black lines on samples denoting fractures, red dotted lines serving as centerlines, and the blue dotted line acting as a thin natural crack line for fracture activation.

Based on photographic analysis and the consideration of various failure characteristics reported in the research of the above-mentioned authors, different failure patterns at post Brazilian tensile strength testing were identified to be associated with the Eagle Ford and Wolfcamp shale samples. These failure patterns were defined to consist of (1) single centralized fracture (SCF) driven by the action of tensile splitting, (2) single non-centralized fracture (SNCF), (3) dual non-centralized fracture (DNCF), (4) multiple non-centralized fracture (MNCF), and (5) natural crack activated fracture (NCAF) caused by the presence of microcrack or thin fracture line on the sample. Examples of centralized, non-centralized, and natural crack activated fractures are shown in (Figure 15). Centralized fracture is concentrated along or nearest to the sample centerline (red dotted line), non-centralized fracture is concentrated away from the centerline, and NCAF is concentrated along the line of natural crack (blue dotted line). Fractures without clear visibility and significant penetration into the samples were excluded in failure pattern classifications. 

**Figure 16:**
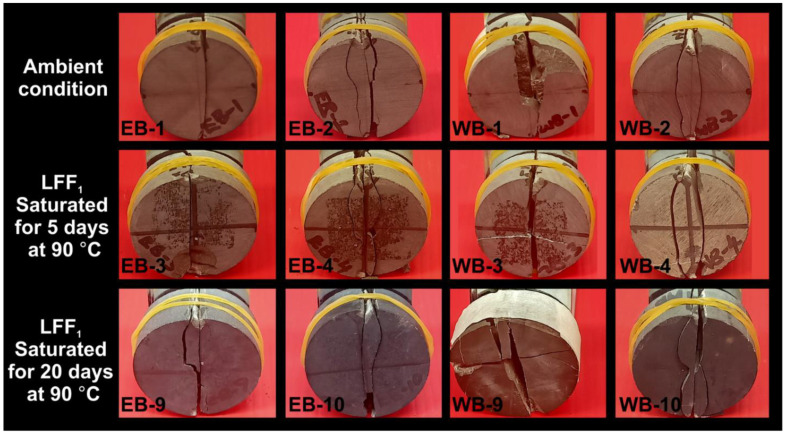
Failure patterns resulting from samples subjected to different treatment conditions prior to BITS testing.

In Table 1, ambient samples EB-1 and WB-1 bearing SNCFs and EB-3 bearing SCF were associated with lower tensile strengths (5.53, 5.86, and 6.21 MPa, respectively), and significant sample surface spalling can be seen on WB-1 and EB-3 (Figure 16). The Wolfcamp shale sample (WB-3) had the highest tensile strength at 11.49 MPa and a deformation pattern of SCF + NCAF, followed by WB-10, with a tensile strength at 11.21 MPa and its failure pattern being SCF + MNCF. It should be noted that only the Wolfcamp samples (WB-3 and WB-9) had NCAFs. The failure pattern images of the samples contained in Table 1 are presented in Figure 16. 

#### 4.4.2. Tensile Strength and Failure Analysis of LFF_2_ Saturated Samples

**Figure 17:**
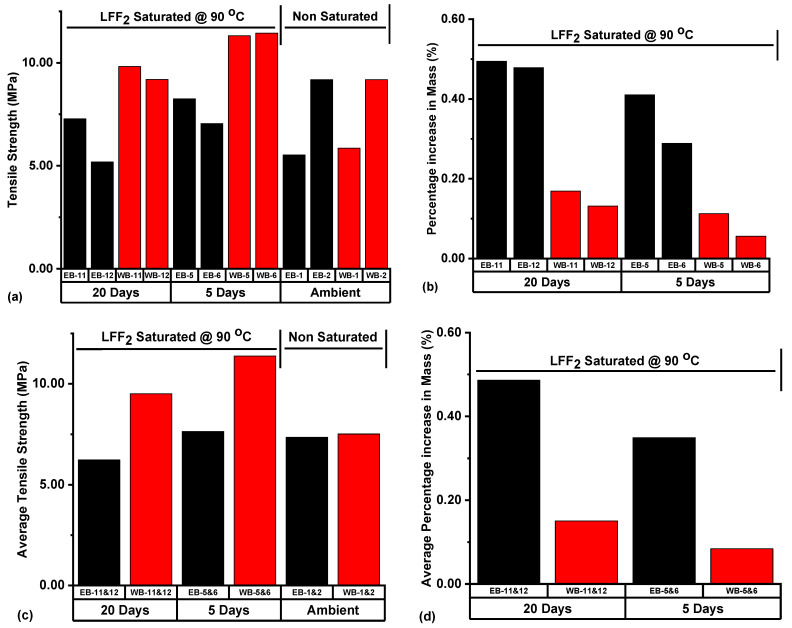
(**a**) Tensile strength, (**b**) Percentage increase in mass, (**c**) Average tensile strength (ATS), and (**d**) Average percentage increase in mass (APIM) distributions of samples subjected to diverse treatment conditions prior to BITS testing. The notes contained in Figure 14 still apply.

The Wolfcamp sample WB-6 at a percentage increase in mass (0.06%) had the highest tensile strength (11.44 MPa), seconded by WB-5 at 11.32 MPa, with a percentage increase in mass of 0.11% based on LFF_2_ saturation at 90 °C (Figure 17a,b). Then, the Eagle Ford shale sample EB-12 had the lowest tensile strength at 5.19 MPa, with a percentage increase in mass of 0.48%. As evident in Figure 17a,b, the Eagle Ford samples, in comparison to the Wolfcamp samples, were more associated with higher values of percentage increase in mass and lower values of tensile strength. 

Figure 17c represented the ATSs of sample sets subjected to different treatment conditions prior to BITS testing. ATS increase was recorded from 7.52 MPa, for ambient Wolfcamp samples WB-1 & 2, to 11.38 MPa, for samples WB-5 & 6. This represented an increase of 51.33% in the ATS following LFF_2_ saturation at 90 °C for 5 days. WB-11 & 12 saturated with LFF_2_ at 90 °C for 20 days recorded an ATS value of 9.50 MPa. This represented an increase of 26.33% with reference to the ATS of ambient samples WB-1 & 2. LFF_2_ saturation of the Wolfcamp samples WB-5 & 6 for 5 days and WB-11 & 12 for 20 days at 90 °C led to 51.33 and 26.33% increases in ATS, respectively. These increases were associated with an APIM value of 0.08% for WB-5 & 6 under 5 days and of 0.15% for WB-11 & 12 under 20 days of LFF_2_ saturation at 90 °C (Figure 17d). It should be noted that, at the lower value of APIM, the ATS was higher at 11.38 MPa for WB-5 & 6, and then, at the higher value of APIM, the ATS was at a lower value (9.50 MPa) for WB-11 & 12. This implied that the higher absorption of LFF_2_ at 90 °C, as reflected by the APIM value (0.15%), induced ATS lowering tendencies on WB-11 & 12 in comparison to the ATS of WB-5 & 6. Thus, the higher fluid saturation level has the potential of gradual reduction of the LFF_2_ adhesive property, weakening the cohesive bonding of rock materials, and, in effect, lead to some tensile strength reductions. 

In the case of the Eagle Ford samples, an ATS increase was recorded from 7.36 MPa, for ambient samples EB-1 & 2, to 7.65 MPa, for EB-5 & 6, under LFF_2_ saturation at 90 °C for 5 days (Figure 17c). This represented an increase of 3.94%, and the associated APIM value was at 0.35% (Figure 17d). The ATS value for EB-11 & 12 under LFF_2_ saturation at 90 °C for 20 days was at 6.23 MPa; this represented an ATS decrease of 15.35% with reference to the ATS of ambient samples and a decrease of 18.56% with reference to the ATS of EB-5 & 6. The decrease in ATS, as associated with samples EB-11 & 12, can be attributed to the higher value of APIM (0.49%) in comparison to that of EB-5 & 6 (0.35%) (Figure 17d). It can also be seen that, at a higher APIM value owing to LFF_2_ saturation at 90 °C, the shale samples experienced a lower value of ATS. The higher APIM reflected the higher LFF_2_ saturation of samples’ accessible pores and can possibly reduce the adhesiveness of LFF_2_, weaken grain bonding, and cause the gradual lowering of samples’ ATS. Then, under a lower value of APIM, the LFF_2_ saturation of pores will provide more impactful bonding effects on shale grains, given that more sites are accessible for LFF_2_ adhesion, thereby boosting the samples’ ATS, as evidenced in Figure 17c,d.

**Figure 18:**
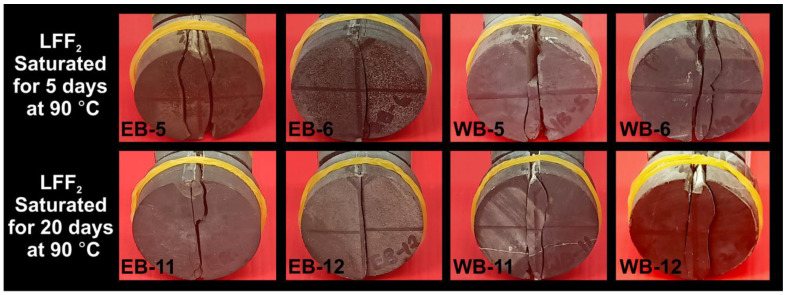
Tensile failure patterns that resulted from the samples subjected to LFF_2_ saturation at 90 °C for the periods of 5 and 20 days prior to BITS testing.

In Table 2, samples with high tensile strengths are associated with either non-centralized fractures or a combination of centralized and non-centralized fractures. For instance, WB-6 at a tensile strength of 11.44 MPa had the failure pattern SCF + SNCF, WB-5 with a tensile strength of 11.32 MPa had the failure pattern SCF + SNCF, WB-11 with a tensile strength of 9.82 MPa had the failure pattern DNCF, and the Eagle Ford sample EB-12, with the lowest tensile strength (5.19 MPa), recorded the failure pattern SCF. Figure 18 represented the tensile failure patterns that resulted from the BITS testing of LFF_2_ saturated samples at 90 °C for the periods of 5 and 20 days, and the presence of a thin natural crack line can be spotted on sample WB-11. 

#### 4.4.3. Tensile Strength and Failure Analysis of Reservoir Temperature Conditioned Samples 

**Figure 19:**
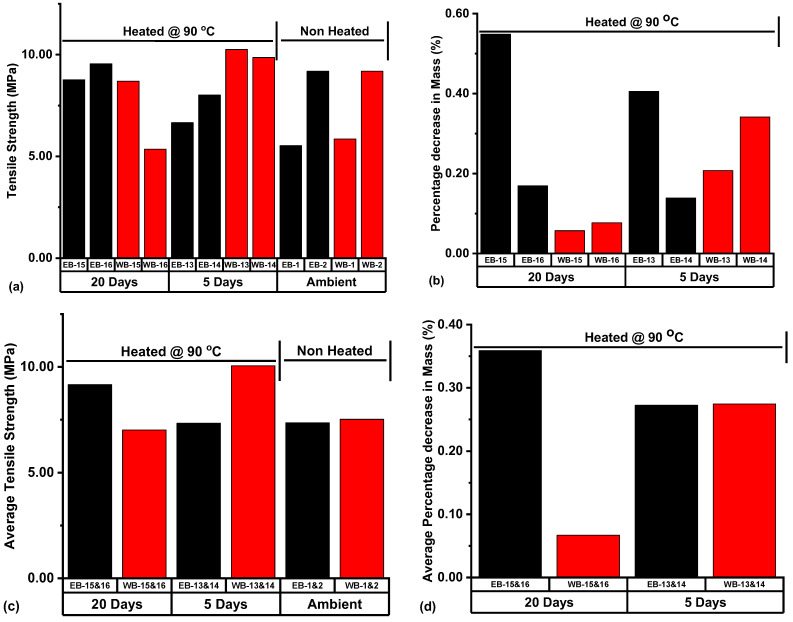
(**a**) Tensile strength, (**b**) Percentage decrease in mass, (**c**) Average tensile strength (ATS), and (**d**) Average percentage decrease in mass (APDM) distributions of samples subjected to different treatment conditions prior to BITS testing. Notes contained in Figure 14 still apply.

Figure 19a represented the tensile strength behaviors of Eagle Ford and Wolfcamp shale samples under different treatment conditions prior to BITS testing. In Figure 19b, the percentage decreases in mass resulting from the thermal exposure of the samples are presented. Among the Wolfcamp shale samples exposed to 90 °C, WB-13 for 5 days and WB-16 for 20 days had the highest and lowest values of tensile strength at 10.25 and 5.36 MPa, respectively. The percentage decreases in mass were 0.21% for WB-13 and 0.08% for WB-16 (Figure 19b). This implied that more pores were accessible to water phase dehydration in WB-13 exposed to 90 °C for 5 days, thereby causing a higher reduction in sample mass, while fewer pores were accessible to water phase dehydration in WB-16 exposed to 90 °C for 20 days. 

Among the Eagle Ford samples exposed to 90 °C, EB-15 on 20 days of thermal exposure had the highest percentage decrease in mass at 0.55%, and EB-14 on 5 days of thermal exposure recorded the lowest percentage decrease in mass (0.14%) (Figure 19b). The tensile strengths associated with these samples were 8.77 and 8.02 MPa for EB-15 and EB-14, respectively. Among the Wolfcamp samples exposed to 90 °C, WB-14 on thermal exposure of 5 days and WB-15 on thermal exposure of 20 days had the highest and lowest percentage decreases in mass (0.34 and 0.06%, respectively) (Figure 19b). The associated tensile strengths were 9.86 MPa for WB-14 and 8.69 MPa for WB-15 (Figure 19a). The samples with higher percentage losses in mass were associated with higher values of tensile strength. 

In Figure 19c,d, the respective ATS and average percentage decrease in mass (APDM) arising from Eagle Ford and Wolfcamp samples subjected to different treatment conditions prior to BITS testing are represented. The Wolfcamp shale samples WB-13 & 14, exposed to 90 °C for a period of 5 days, had an ATS value of 10.06 MPa, which represented an ATS increase of 33.78% with reference to the ATS of ambient samples WB-1 & 2. The associated APDM of WB-13 & 14 was 0.27% (Figure 19d). The Wolfcamp samples WB-15 & 16, heated for 20 days at 90 °C and associated with an APDM of 0.07%, had an ATS value of 7.02 MPa. This represented an ATS decrease of 6.65% with reference to that of ambient samples WB-1 & 2. The Eagle Ford samples EB-13 & 14, exposed to thermal conditions of 90 °C for 5 days and associated with an APDM of 0.27%, had an ATS value of 7.34 MPa, which represented an ATS decrease of 0.27% with reference to the ATS of ambient samples EB-1 & 2. Equally, the Eagle Ford samples EB-15 & 16, under thermal conditions of 90 °C for 20 days and associated with an APDM value of 0.36%, had an ATS value of 9.16 MPa. This represented an ATS increase of 24.46% with reference to the ATS of ambient samples EB-1 & 2. 

5 days of thermal exposure to 90 °C for the Wolfcamp samples WB-13 & 14, associated with an APDM value of 0.27%, led to 33.78% increase in ATS with reference to the ATS of ambient samples WB-1 & 2. Then, for WB-15 & 16 subjected to 20 days of thermal exposure to 90 °C and associated with an APDM of 0.07%, 6.65% decrease in ATS with reference to the ATS of the ambient samples resulted. This implied that more pores were accessible for water phase dehydration in samples WB-13 & 14, as reflected by the higher APDM in comparison to that of WB-15 & 16. The higher water phase dehydration could have created an avenue for the likely effective dilation of calcite minerals. This is sufficient to cause grains’ compaction and the closure of pores and micro-fractures, thereby resulting in the ATS boosting of WB-13 & 14. This equally can be attributed to the case of Eagle Ford samples EB-15 & 16 under 20 days of thermal exposure to 90 °C, with a higher APDM (0.36%) and a recorded ATS increase of 24.46% with reference to the ATS of ambient samples EB-1 & 2. Yavuz et al. [61] reported that the thermal exposure of monomineralic rock (calcite-rich) is liable to cause the dilation of calcite minerals. Idris [49], Rao et al. [62], and Sirdesai et al. [63] equally reported that the thermal exposure of rock sample can cause rock mineral expansion, consequent grain compaction, and the closure of pores and pre-existing fissures. These internal activities will eventually boost the strength of rock materials.

**Figure 20:**
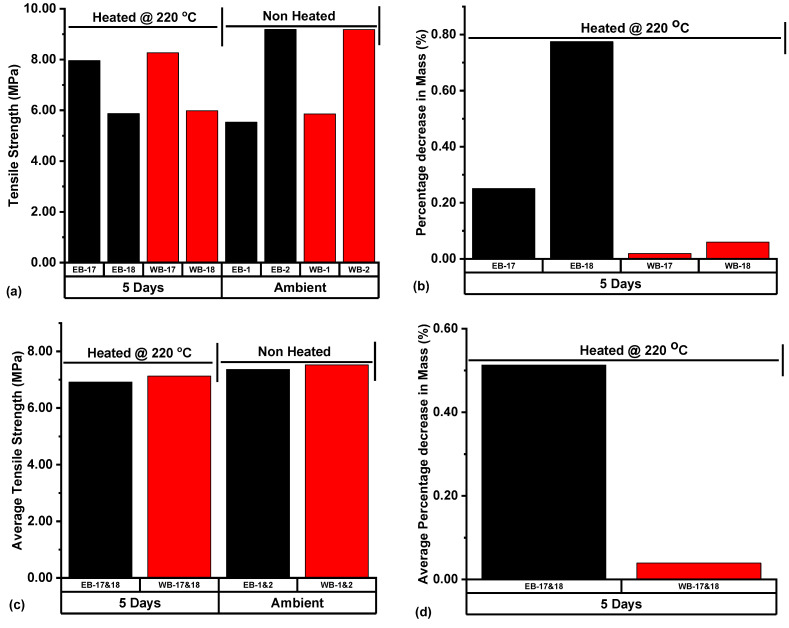
(**a**) Tensile strength, (**b**) Percentage decrease in mass, (**c**) Average tensile strength (ATS), and (**d**) Average percentage decrease in mass (APDM) resulting from samples subjected to different treatment conditions prior to BITS testing. Notes contained in Figure 14 still apply.

Eagle Ford and Wolfcamp shale samples were exposed to 220 °C, considered as the upper limit of reservoir temperature, for a period of 5 days, with the resulting samples’ tensile strengths compared to those of ambient samples (Figure 20a). The associated percentage decreases in mass resulting from the thermally exposed samples are represented in Figure 20b. Eagle Ford sample EB-18, subjected to 5 days of thermal exposure to 220 °C recorded a percentage decrease in mass of 0.77% and an associated tensile strength of 5.87 MPa. EB-17 had a percentage decrease in mass of 0.25% and a resulting tensile strength at 7.95 MPa. The Wolfcamp sample WB-18 under the same heat treatment condition had a percentage decrease in mass of 0.06% and a tensile strength of 5.98 MPa. Sample WB-17 recorded a percentage decrease in mass of 0.02% and a tensile strength at 8.27 MPa (Figure 20a,b). The higher values of percentage decrease in mass resulting from 5 days of thermal exposure of the Eagle Ford and Wolfcamp shale samples to 220 °C were associated with lower tensile strength values. 

The ATS value of Eagle Ford samples EB-17 & 18 after 5 days of thermal exposure to 220 °C was 6.91 MPa, and the associated APDM was 0.51%. This ATS value represented a decrease of 6.11% with reference to that of ambient samples EB-1 & 2, at an ATS of 7.36 MPa (Figure 20c,d). In the case of Wolfcamp samples WB-17 & 18, with an APDM at 0.04%, the ATS was 7.12 MPa and thus represented a decrease of 5.32% with reference to that of ambient samples WB-1 & 2. These results demonstrated that 220 °C thermal exposure of the Eagle Ford and Wolfcamp samples led to decreases in the ATS by 6.11 and 5.32%, respectively. This implied that, under this temperature condition, there is a possible formation of new-microcracks in the samples, thus boosting the porosity and inducing strength reduction conditions on the samples. Idris [49], Rao et al. [62], and Sirdesai et al. [63] equally reported that rock minerals are associated with different thermal expansion coefficients, and once these coefficients are exceeded, the formation of new-microcracks is inevitable and will consequently lead to the strength weakening of rock materials. 

**Figure 21:**
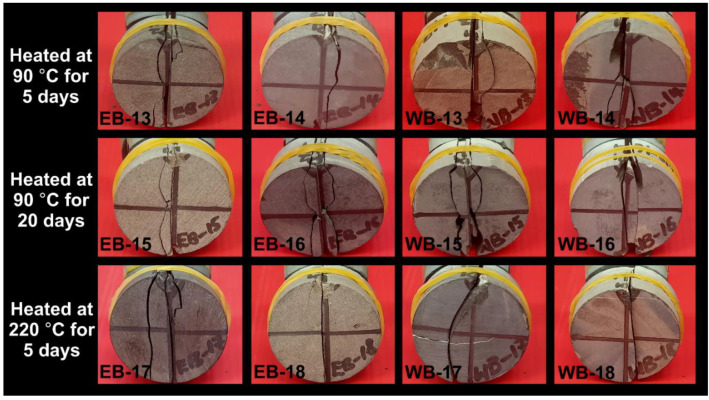
Image representation of tensile failure patterns resulting from Eagle Ford and Wolfcamp shale samples exposed to different heat treatment conditions prior to BITS testing.

The failure patterns that resulted from Eagle Ford and Wolfcamp samples subjected to different heat treatment conditions prior to BITS testing are classified and presented in Table 3. The failure patterns of samples with very low values of tensile strength included SCF + SNCF for WB-16 at a tensile strength of 5.36 MPa, SCF for EB-18 at a tensile strength of 5.87 MPa, and SNCF for WB-18 at a tensile strength of 5.98 MPa. The Wolfcamp sample WB-13 had the highest tensile strength, with a failure pattern of SCF + SNCF, followed by WB-14 at a tensile strength of 9.86 MPa and having a failure pattern of SCF + DNCF. The image representation of samples’ failure patterns is contained in Figure 21. Single fractures are more dominant in the 220 °C exposed samples, while multiple fractures dominated the samples exposed to 90 °C prior to BITS testing.

#### 4.4.4. Failure Crack Length Analysis

**Figure 22:**
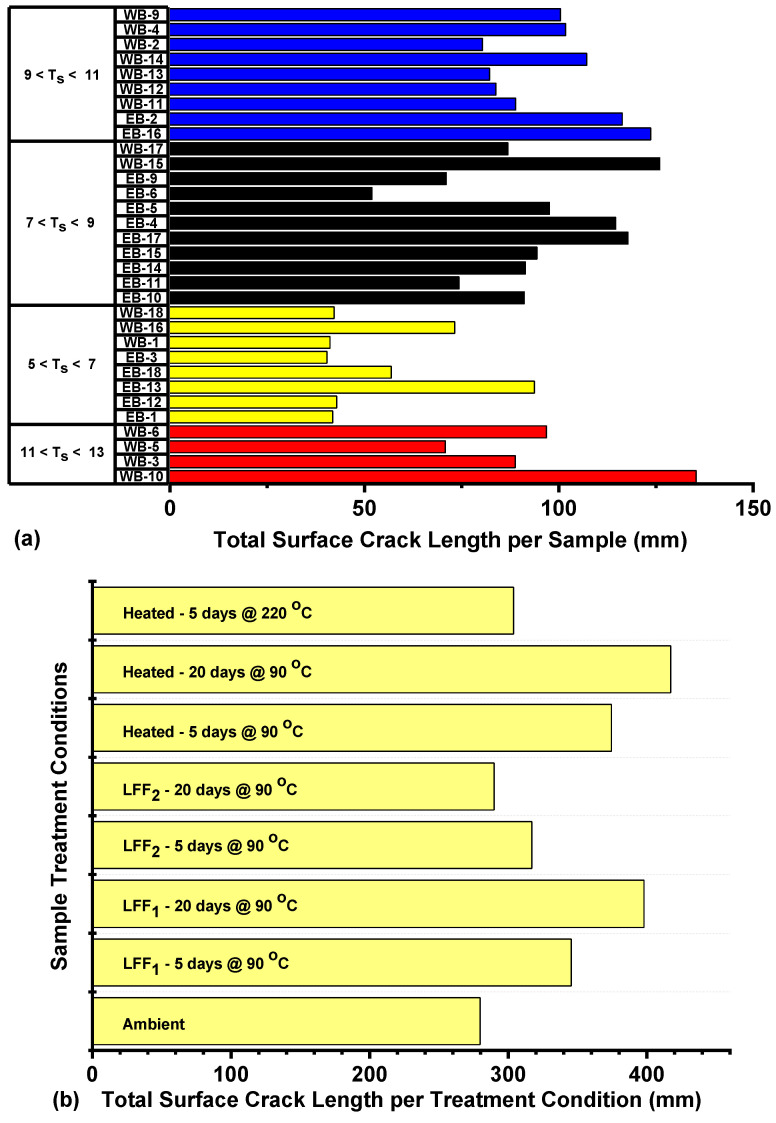
(**a**) Total surface crack length per sample for Groups 1–4 based on respective samples’ tensile strengths, and (**b**) Total surface crack length per treatment condition for the various sample sets.

**Figure 23:**
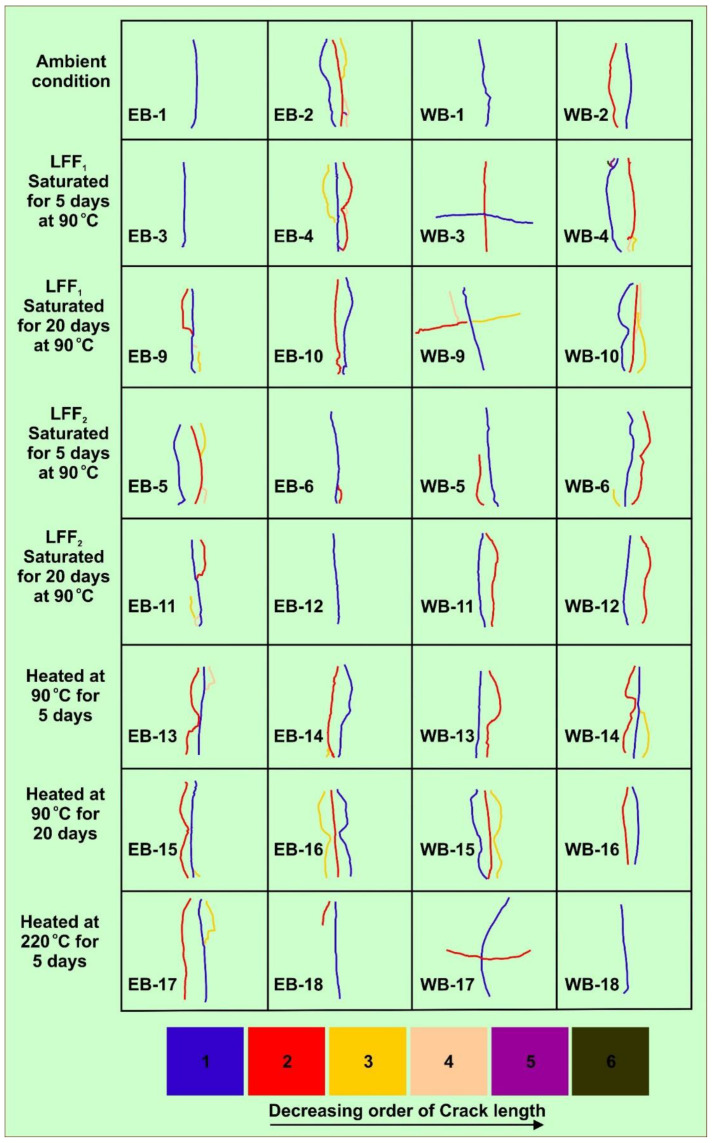
Traces of crack/fracture patterns resulting from various sample sets (ambient, thermally conditioned LFF_1 & 2_ saturated, and heat treated at 90 and 220 °C) at post BITS testing, and these traces were used in sample surface crack length quantifications.

The failure pattern images (Figure 16, Figure 18, Figure 21) of the 32 samples at post BITS testing were subjected to crack length analysis using the line tracing and measurement tools of CorelDRAW Graphics Suite, 2021. Most visible cracks or fractures resulting from BITS testing and present on samples’ surfaces were traced and measured to account for surface crack lengths. The crack lengths on each sample surface were summed up to determine the total surface crack length per sample (Figure 22a). Then, based on samples’ treatment conditions, the total surface crack length per treatment condition (Figure 22b) was quantified. The tensile strengths of the 32 samples were classified into (a) Group 1 (5 < T_s_ < 7), (b) Group 2 (7 < T_s_ < 9), (c) Group 3 (9 < T_s_ < 11), and (d) Group 4 (11 < T_s_ < 13), with T_s_ denoting the tensile strength in MPa. In Figure 22a, the highest total surface crack length per sample was 135.31 mm for sample WB-10 in Group 4, followed by a total surface crack length at 125.97 mm for WB-15 in Group 2, and the lowest total surface crack length was 40.36 mm for sample EB-3 in Group 1 (Figure 22a). Samples of high tensile strengths are more likely to be associated with more surface crack lengths in comparison to samples of low tensile strengths. The presence of several cracks or fractures will drive favorable fracture network formation towards efficient shale gas production through hydraulic fracturing applications. Based on the treatment conditions of the samples, heat treated samples at 90 °C for 20 days had the highest overall surface crack length at 417.21 mm, followed by LFF_1_ saturated samples at 90 °C for 20 days at 397.92 mm, and ambient samples at 279.69 mm being the lowest (Figure 22b). These values are justified by the presence of more fractures at post BITS testing of heat treated samples and LFF_1_ saturated samples at 90 °C for 20 days than on ambient samples, as shown in Figure 23. 

### 4.5. Discussion of Results

The FESEM-EDX system facilitated the mineralogical characterization and elemental distribution analysis of Eagle Ford and Wolfcamp samples. The rich distribution of Ca, C, and O supported the dominant presence of calcite (CaCO_3_) in the Eagle Ford and Wolfcamp shales. Denser distributions of Si supported the more dominant presence of quartz in Eagle Ford than in Wolfcamp shale. The FESEM images showcased variabilities in the mineralogical structures of Eagle Ford and Wolfcamp shales, and these variabilities were most evident in the Eagle Ford shale. The Eagle Ford shale was of higher porosity and permeability at 2.95% and 2.50 × 10^−5^ µm^2^, while those of the Wolfcamp were at 1.61% and 1.64 × 10^−5^ µm^2^. The pore volume distribution of the Eagle Ford was greater than that of Wolfcamp, given their respective cumulative pore volumes at 13.62 mm^3^/g and 9.03 mm^3^/g. Owing to these, more pores were accessible in the LFF saturation and heat treatment of Eagle Ford samples than in Wolfcamp samples. 

LFF_1_ saturation of the Eagle Ford samples under 5 days resulted in a 3.13% decrease in ATS with reference to the ATS of the ambient Eagle Ford samples, but under 20 days, a 6.79% increase in ATS was recorded. The APIM under 5 days of LFF_1_ saturation at 90 °C was 0.55%, and under 20 days it was 0.34% (Figure 14d). In the case of 5 days of LFF_2_ saturation at 90 °C, an ATS increase of 3.94% was recorded and the associated APIM was at (0.35%). Then. under 20 days of saturation, a 15.35% decrease in ATS resulted, along with an APIM value at 0.49% (Figure 17c,d). LFF_1_ saturation of the Wolfcamp samples at 90 °C for 5 days led to a 48.01% increase in ATS, and under 20 days it led to an ATS increase of 40.29% with reference to the ATS of ambient samples (Figure 14c). The associated APIM were 0.12 and 0.19% under 5 and 20 days, respectively (Figure 14d). ATS increases of 51.33 and 26.33% were recorded in the LFF_2_ saturation of Wolfcamp samples at 90 °C for 5 and 20 days, respectively, with reference to the ATS of the ambient samples (Figure 17c). The associated APIM under 5 and 20 days of LFF_2_ saturation at 90 °C were 0.08 and 0.15%, respectively (Figure 17d). 

Based on the above values of ATS and APIM resulting from the LFF saturations of Eagle Ford and Wolfcamp samples, it is clear that at higher APIM values, the shale samples experienced lower values of ATS. The higher APIM reflected the higher LFF saturation of samples’ accessible pores. This will gradually reduce LFF adhesiveness, weaken grain bonding, and lower samples’ ATS. At lower values of APIM, the LFF saturation of pores possibly provided a more impactful bonding effect on shale grains due to the stronger adhesion at low fluid saturation, thereby boosting samples’ ATS values. The ATS increases associated with the Wolfcamp samples were 51.33, 48.01, 40.29, and 26.33%, while those of the Eagle Ford samples were 6.79 and 3.94%. The Eagle Ford samples equally recorded ATS decreases of 15.35 and 3.13%. These implied that the ATS behaviors of the Wolfcamp samples were more consistent than those of the Eagle Ford samples. This could be a result of the Eagle Ford shale’s heterogeneous complexity, as reported by Milliken et al. [64] and equally supported by the FESEM images contained in Figure 8b,d. 

The samples of Eagle Ford and Wolfcamp exposed to reservoir temperature conditions of 90 °C for 5 and 20 days were evaluated based on ATS and APDM. It was revealed that higher values of APDM led to ATS increases of the Eagle Ford and Wolfcamp samples, while ATS decreases resulted from lower values of APDM. Higher APDM implied that more pores were accessible for water phase dehydration. This provided an avenue for the possibility of more effective calcite dilation, triggering grain compaction and the closure of pores and micro-fractures, thus boosting the ATS of the samples. 

In the thermal exposure of the Eagle Ford and Wolfcamp shale samples to 220 °C for 5 days, the Wolfcamp shales associated with an APDM of 0.04% had an ATS decrease of (5.32%), and the Eagle Ford shales associated with an APDM of 0.51% had an ATS decrease of 6.11% with reference to the ATS of their respective ambient samples. ATS decreases resulted from the samples’ exposure to 220 °C. This high temperature condition has the tendency to induce the creation of new microcracks, boost porosity, and consequently lead to tensile strength reductions. 

Several results have been generated from this research, ranging from mineralogical characterization, elemental distribution analysis, porosity–permeability evaluations, ATS analysis under diverse conditions, tensile failure classifications to crack length analysis. The dominant presence of calcite and quartz minerals in Eagle Ford shale will promote shale brittle failure and discourage plastic deformation. LFF driven hydraulic fracturing will lead to more fluid absorption into Eagle Ford shale owing to its higher porosity and permeability characteristics than that of Wolfcamp. Shale–LFF interactions during hydraulic fracturing may not lead to a drastic loss of shale tensile strength. Eagle Ford and Wolfcamp shales’ fracturing using LFF will result in the initiation and propagation of diverse tensile failure patterns. These will help to promote the formation of complex fracture networks, thereby encouraging porosity–permeability transformation for efficient and sustainable shale gas productions in the Eagle Ford and Wolfcamp shales. 

## 5. Conclusions

The FESEM-EDX-Mapping analysis established that Eagle Ford and Wolfcamp shales had rich distributions of Ca, C, and O in support of the dominant presence of calcite minerals. MIP is a competent technique and aided the quantitative evaluation of porosity and permeability, which were found to be higher in Eagle Ford shale than in Wolfcamp shale. Porosities and permeabilities were 2.95% and 2.50 × 10^−5^ µm^2^ for the Eagle Ford shale and 1.61% and 1.64 × 10^−5^ µm^2^ for the Wolfcamp shale. 

The thermally conditioned LFF saturation of Eagle Ford and Wolfcamp shales resulted in ATS increases ranging from 26.33–51.33% for the Wolfcamp samples, while the Eagle Ford samples had ATS increases of 3.94 and 6.79% and ATS decreases of 3.13 and 15.35%. The adhesiveness of LFF necessitated the possible compaction of shale grains to boost the ATS of shale samples at lower APIM. Higher values of APIM likely reduced LFF adhesiveness, weakened grain bonding, and necessitated the gradual lowering of ATS. 

The reservoir temperature conditioning of Eagle Ford and Wolfcamp samples at 90 °C resulted in respective ATS increases of 24.46 and 33.78% at higher values of APDM. Higher values of APDM reflected higher levels of water phase dehydration in accessible pores. This provided an avenue for possible calcite dilation, triggering grain compaction and the closure of pores and micro-fractures to boost the ATS of samples. Eagle Ford and Wolfcamp shales under thermal exposure to 220 °C recorded ATS decreases of 6.11 and 5.32%, respectively. The likely new microcracks’ initiation tendencies at this temperature have the capacity to induce samples’ ATS reductions. 

## Figures and Tables

**Table 1:** Tensile failure analysis of samples with indications of their treatment conditions prior to BITS testing. Identified failure patterns included SCF, SNCF, DNCF, MNCF, and NCAF.

S/No.	Sample ID	Treatment Conditions	Tensile Strength (MPa)	Failure Type	Remarks
1	EB-1	Ambient samples	5.53	SNCF	Lowest tensile strength
2	EB-2		9.18	SCF + DNCF	Non
3	WB-1		5.86	SNCF	Spalling at upper section
4	WB-2		9.18	DNCF	Non
5	EB-3	LFF_1_ saturated for 5 days at 90 °C	6.21	SCF	Spalling at lower section
6	EB-4		8.05	SCF + DNCF	Non
7	WB-3		11.49	SCF + NCAF	Highest tensile strength
8	WB-4		10.76	DNCF	Thin crack line at lower section
9	EB-9	LFF_1_ saturated for 20 days at 90 °C	7.86	SCF + SNCF	Non
10	EB-10		7.85	SCF + SNCF	Deep grey coloration
11	WB-9		9.89	SCF + SNCF + NCAF	Thin lines of natural cracks
12	WB-10		11.21	SCF + MNCF	Second highest tensile strength

**Table 2:** Tensile failure analysis of samples subjected to LFF_2_ saturation at 90 °C for the periods of 5 and 20 days prior to BITS testing. Identified failure patterns included SCF, SNCF, DNCF, and MNCF.

S/No.	Sample ID	Treatment Conditions	Tensile Strength (MPa)	Failure Type	Remarks
13	EB-5	LFF_2_ saturated for 5 days at 90 °C	8.25	MNCF	Non
14	EB-6		7.05	SNCF	Non
15	WB-5		11.32	SCF + SNCF	Second highest tensile strength
16	WB-6		11.44	SCF + SNCF	Highest tensile strength
17	EB-11	LFF_2_ saturated for 20 days at 90 °C	7.28	SCF + SNCF	Non
18	EB-12		5.19	SCF	Lowest tensile strength
19	WB-11		9.82	DNCF	Natural fracture line at lower section
20	WB-12		9.19	DNCF	Non

**Table 3:** Tensile failure classifications for samples subjected to reservoir temperature controlled conditions of 90 °C for 5 and 20 days and 220 °C for 5 days prior to BITS testing. Identified failure patterns included SCF, SNCF, DNCF, and NCAF.

S/No.	Sample ID	Treatment Conditions	Tensile Strength (MPa)	Failure Type	Remarks
21	EB-13	Heated at 90 °C for 5 days	6.66	SCF + SNCF	Minor fracture at upper section
22	EB-14		8.02	SCF + SNCF	Non
23	WB-13		10.25	SCF + SNCF	Spalling at upper section
24	WB-14		9.86	SCF + DNCF	Spalling at sample surface
25	EB-15	Heated at 90 °C for 20 days	8.77	SCF + SNCF	Non
26	EB-16		9.55	SCF + DNCF	Non
27	WB-15		8.69	SCF + DNCF	Non
28	WB-16		5.36	SCF + SNCF	Non
29	EB-17	Heated at 220 °C for 5 days	7.95	SCF + DNCF	Non
30	EB-18		5.87	SCF	Non
31	WB-17		8.27	SNCF + NCAF	Natural crack activation oriented
32	WB-18		5.98	SNCF	Thin natural fracture line

## Data Availability

Data can be made available based on request; however very reasonable amount of data is already contained in the manuscript.

## Supplementary Materials

The following are available online at https://www.mdpi.com/article/10.3390/polym14122417/s1, Figure S1: Cylindrically shaped vertical plugs of Eagle Ford and Wolfcamp shales used in construction of disc-shaped samples for Brazilian Indirect Tensile Strength testing, Figure S2: (a) FESEM-EDX set-up for mineralogical characterization and elemental composition analysis, (b) 140 & 240 Series Mercury Porosimeters for porosity-permeability analysis, and (c) Sputter coater for metallic coating of sample splits to aid conductive layering, Figure S3: Experimental set-up of (a) Model 9B 5-Spindle Multimixer to facilitate homogeneous dissolution of chemical additives, (b) Binder oven for thermal conditioning of samples at 90 °C, and (c) Carbolite chamber furnace for thermal conditioning of samples at 220 °C, Figure S4: Brazilian disc-shaped samples of Eagle Ford and Wolfcamp shales placed uprightly in pairs in borosilicate glass containers and filled with linear fracturing fluids of polymer concentrations at 0.65 and 2.50% in preparation for thermal conditioning. The borosilicate glass containers were covered with aluminum foils and afterwards were tightly sealed with metallic lids before placement for thermal conditioning, Figure S5: Brazilian indirect tensile strength testing equipment set up with disc-shaped sample of Eagle Ford shale held between loading platens with diameter-concentrated compressional loading applied to failure.

## References

[1] Acosta J.C., Dang S., Curtis M., Sondergeld C., Rai C. Fracturing Fluids Effect on Mechanical Properties in Shales. Proceedings of the 8th Unconventional Resources Technology Conference.

[2] Gandossi L. (2013). An Overview of Hydraulic Fracturing and Other Formation Stimulation Technologies for Shale Gas Production.

[3] Gu M., Mohanty K.K. (2015). Rheology of polymer-free foam fracturing fluids. J. Pet. Sci. Eng..

[4] Barati R., Liang J.-T. (2014). A review of fracturing fluid systems used for hydraulic fracturing of oil and gas wells. J. Appl. Polym. Sci..

[5] Chauhan G., Verma A., Doley A., Ojha K. (2018). Rheological and breaking characteristics of Zr-crosslinked gum karaya gels for high-temperature hydraulic fracturing application. J. Pet. Sci. Eng..

[6] Aliu A.O., Guo J., Wang S., Zhao X. (2016). Hydraulic fracture fluid for gas reservoirs in petroleum engineering applications using sodium carboxy methyl cellulose as gelling agent. J. Nat. Gas Sci. Eng..

[7] Al-Hajri S., Negash B.M., Rahman M., Haroun M., Al-Shami T.M. (2022). Perspective Review of Polymers as Additives in Water-Based Fracturing Fluids. ACS Omega.

[8] Al-Muntasheri G. A Critical Review of Hydraulic Fracturing Fluids over the Last Decade. Proceedings of the SPE Western North America and Rocky Mountain Joint Regional Meeting.

[9] Lufeng Z., Fujian Z., Shicheng Z., Zhun L., Jin W., Yuechun W. (2019). Evaluation of permeability damage caused by drilling and fracturing fluids in tight low permeability sandstone reservoirs. J. Pet. Sci. Eng..

[10] Akrad O.M., Miskimins J.L., Prasad M. The Effects of Fracturing Fluids on Shale Rock Mechanical Properties and Proppant Embedment. Proceedings of the SPE Annual Technical Conference and Exhibition.

[11] Duan K., Kwok C. (2015). Discrete element modeling of anisotropic rock under Brazilian test conditions. Int. J. Rock Mech. Min. Sci..

[12] Li H., Lai B., Liu H.-H., Zhang J., Georgi D. (2017). Experimental Investigation on Brazilian Tensile Strength of Organic-Rich Gas Shale. SPE J..

[13] Ma T., Peng N., Zhu Z., Zhang Q., Yang C., Zhao J. (2018). Brazilian Tensile Strength of Anisotropic Rocks: Review and New Insights. Energies.

[14] Jaeger J.C., Cook N.G.W., Zimmerman R.W. (2007). Fundamentals of Rock Mechanics.

[15] (2001). Splitting Tensile Strength of Intact Rock Core Specimens 1.

[16] Chen C.S., Hsu S.C. (2001). Measurement of Indirect Tensile Strength of Anisotropic Rocks by the Ring Test. Rock Mech. Rock Eng..

[17] Lai B.T., Li H., Liu H.H., Zhang J.L., Georgi D. Brazilian Tensile Strength Test of Orgainc-Rich Shale. Proceedings of the SPE Abu Dhabi International Petroleum Exhibition and Conference.

[18] Lin W. Mechanical Properties of Mesaverde Shale and Sandstone at High Pressure. Proceedings of the AIRAPT Conference on High Pressure and 19 EHPRG Conference.

[19] Chong K.P., Chen J.L., Dana G.F., Weber J.A. (1984). Indirect and Direct Tensile Behaviour of Devonian Oil Shales.

[20] Mokhtari M., Honarpour M.M., Tutuncu A.N., Boitnott G.N. Acoustical and Geomechanical Characterization of Eagle Ford Shale—Anisotropy, Heterogeneity and Measurement Scale. Proceedings of the SPE Annual Technical Conference and Exhibition.

[21] Gao Q., Tao J., Hu J., Yu X. (2015). Laboratory study on the mechanical behaviors of an anisotropic shale rock. J. Rock Mech. Geotech. Eng..

[22] He J., Afolagboye L.O. (2017). Influence of layer orientation and interlayer bonding force on the mechanical behavior of shale under Brazilian test conditions. Acta Mech. Sin. Xuebao.

[23] Hou B., Zeng Y., Fan M., Li D. (2018). Brittleness Evaluation of Shale Based on the Brazilian Splitting Test. Geofluids.

[24] Simpson N.D.J., Stroisz A., Bauer A., Vervoort A., Holt R.M. (2014). Failure mechanics of anisotropic shale during Brazilian tests. 48th US Rock Mechanics/Geomechanics Symposium Proceedings.

[25] Wang J., Xie L., Xie H., Ren L., He B., Li C., Yang Z., Gao C. (2016). Effect of layer orientation on acoustic emission characteristics of anisotropic shale in Brazilian tests. J. Nat. Gas Sci. Eng..

[26] Yang S.-Q., Yin P.-F., Huang Y.-H. (2019). Experiment and Discrete Element Modelling on Strength, Deformation and Failure Behaviour of Shale Under Brazilian Compression. Rock Mech. Rock Eng..

[27] Vernik L., Nur A. (1992). Ultrasonic velocity and anisotropy of hydrocarbon source rocks. Geophysics.

[28] Amadei B., Jonsson T. Tensile strength of anisotropic rocks measured with the splitting tension test. Proceedings of the 12th Southeastern Conference Theoretical and Applied Mechanics.

[29] Chen C.-S., Pan E., Amadei B. (1998). Determination of deformability and tensile strength of anisotropic rock using Brazilian tests. Int. J. Rock Mech. Min. Sci..

[30] Lekhnitskii S.G., Tsai S.W., Cheron T. (1968). Anisotropic Plates.

[31] Claesson J., Bohloli B. (2002). Brazilian test: Stress field and tensile strength of anisotropic rocks using an analytical solution. Int. J. Rock Mech. Min. Sci..

[32] Lee Y.-K., Pietruszczak S. (2015). Tensile failure criterion for transversely isotropic rocks. Int. J. Rock Mech. Min. Sci..

[33] Cho J.-W., Kim H., Jeon S., Min K.-B. (2012). Deformation and strength anisotropy of Asan gneiss, Boryeong shale, and Yeoncheon schist. Int. J. Rock Mech. Min. Sci..

[34] Mighani S., Sondergeld C.H., Rai C.S. (2016). Observations of Tensile Fracturing of Anisotropic Rocks. SPE J..

[35] Yang Z.P., He B., Xie L.Z., Li C., Wang J. (2015). Strength and failure modes of shale based on Brazilian test. Rock Soil Mech..

[36] Yao G., Chen Q., Liu H., Tan Y., Wang L., Du H., Zhu H. (2015). Experiment study on mechanical properties of bedding shale in Lower Silurian Longmaxi shale Southeast Chongqing. Chin. J. Rock Mech. Eng..

[37] ISRM (2007). The Complete ISRM Suggested Methods for Characterization, Testing and Monitoring: 1974–2006, Suggested Methods Prepared by the Commission on Testing Methods, ISRM.

[38] Speight J.G. (2020). Shale Oil and Gas Production Processes.

[39] Ramiro-Ramirez S. (2016). Petrographic and Petrophysical Characterization of the Eagle Ford Shale in La Salle and Gonzales Counties, Gulf Coast Region, Texas. Master’s Thesis.

[40] Jones R. (2019). Nanopetrophysical Characterization of the Wolfcamp A Shale Formation in the Permian Basin of Southeastern New Mexico, USA. Master’s Thesis.

[41] Sutton L. (2014). Permian Basin Geology: The Midland Basin vs. the Delaware Basin Part 2. https://www.enverus.com/blog/permian-basin-geology-midland-vs-delaware-basins/.

[42] Workman S.J. (2013). Integrating Depositional Facies and Sequence Stratigraphy in Characterizing Unconventional Reservoirs: Eagle Ford Shale, South Texas. Master’s Thesis.

[43] Fink J.K. (2013). Hydraulic Fracturing Chemicals and Fluids Technology.

[44] Ali M., Hascakir B. (2016). Water/Rock Interaction for Eagle Ford, Marcellus, Green River, and Barnett Shale Samples and Implications for Hydraulic-Fracturing-Fluid Engineering. SPE J..

[45] Anovitz L.M., Cole D.R. (2015). Characterization and Analysis of Porosity and Pore Structures. Rev. Miner. Geochem..

[46] Clarkson C., Solano N., Bustin R., Bustin A., Chalmers G., He L., Melnichenko Y., Radliński A., Blach T. (2013). Pore structure characterization of North American shale gas reservoirs using USANS/SANS, gas adsorption, and mercury intrusion. Fuel.

[47] Zou C., Zhu R., Tao S., Hou L., Yuan X., Zhang G., Song Y., Niu J., Dong D., Wu X. (2017). Unconventional Petroleum Geology.

[48] Kang Y., Chen M., You L., Li X. (2015). Laboratory Measurement and Interpretation of the Changes of Physical Properties after Heat Treatment in Tight Porous Media. J. Chem..

[49] Idris M.A. (2018). Effects of elevated temperature on physical and mechanical properties of carbonate rocks in South-Southern Nigeria. Min. Miner. Depos..

[50] Saiang C., Miskovsky K. Effect of heat on the mechanical properties of selected rock types—A laboratory study. Harmon. Proceedings of the 12th ISRM International Congress on Rock Mechanics.

[51] Liu S., Xu J. (2015). An experimental study on the physico-mechanical properties of two post-high-temperature rocks. Eng. Geol..

[52] Bailey L., Keall M., Audibert A., Lecourtier J. (1994). Effect of Clay/Polymer Interactions on Shale Stabilization during Drilling. Langmuir..

[53] Grillet A.M., Wyatt N.B., Gloe L.M. (2012). Polymer Gel Rheology and Adhesion. Rheology.

[54] Karakul H. (2018). Effects of drilling fluids on the strength properties of clay-bearing rocks. Arab. J. Geosci..

[55] Kropka J.M., Adolf D.B., Spangler S., Austin K., Chambers R.S. (2015). Mechanisms of degradation in adhesive joint strength: Glassy polymer thermoset bond in a humid environment. Int. J. Adhes. Adhes..

[56] Zosel A. (1985). Adhesion and tack of polymers: Influence of mechanical properties and surface tensions. Colloid Polym. Sci..

[57] Al-Shajalee F., Arif M., Myers M., Tadé M.O., Wood C., Saeedi A. (2021). Rock/Fluid/Polymer Interaction Mechanisms: Implications for Water Shut-off Treatment. Energy Fuels.

[58] Mishra S., Bera A., Mandal A. (2014). Effect of Polymer Adsorption on Permeability Reduction in Enhanced Oil Recovery. J. Pet. Eng..

[59] Basu A., Mishra D.A., Roychowdhury K. (2013). Rock failure modes under uniaxial compression, Brazilian, and point load tests. Bull. Eng. Geol. Environ..

[60] Tavallali A., Vervoort A. (2010). Effect of layer orientation on the failure of layered sandstone under Brazilian test conditions. Int. J. Rock Mech. Min. Sci..

[61] Yavuz H., Demirdag S., Caran S. (2010). Thermal effect on the physical properties of carbonate rocks. Int. J. Rock Mech. Min. Sci..

[62] Rao Q., Wang Z., Xie H., Xie Q. (2007). Influence of Ti 4 + doping on hyperfine field parameters of. J. Cent. South Univ. Technol..

[63] Sirdesai N.N., Singh T.N., Ranjith P.G., Singh R. (2016). Effect of Varied Durations of Thermal Treatment on the Tensile Strength of Red Sandstone. Rock Mech. Rock Eng..

[64] Milliken K.L., Ergene S.M., Ozkan A. (2016). Quartz types, authigenic and detrital, in the Upper Cretaceous Eagle Ford Formation, South Texas, USA. Sediment. Geol..

