# Supplementary material for: Experimental Investigation of Shale Tensile Failure under Thermally Conditioned Linear Fracturing Fluid (LFF) System and Reservoir Temperature Controlled Conditions
Source: Polymers (Basel). 2022 Jun 14;14(12):2417. doi: 10.3390/polym14122417 (PMC9227065; doi:10.3390/polym14122417)
Supplement: Supplementary file 1 [file polymers-14-02417-s001.zip › polymers-1640035-supplementary.pdf]

polymers

MDPI

Supplementary Materials

Experimental Investigation of Shale Tensile Failure under Thermally Conditioned Linear Fracturing Fluid (LFF) System and Reservoir Temperature Controlled Conditions

Cajetan Chimezie Iferobia [1], Maqsood Ahmad [1,*] and Imtiaz Ali [1,2]

[1] Department of Petroleum Engineering, Universiti Teknologi PETRONAS, 32610 Seri Iskandar, Perak Darul Ridzuan, Malaysia; cajetan_17007975@utp.edu.my (C.C.I.); imtiaz_17003333@utp.edu.my (I.A.)
[2] Department of Petroleum and Gas Engineering, BUITEMS, 87300 Quetta, Balochistan, Pakistan
* Correspondence: maqsood.ahmad@utp.edu.my

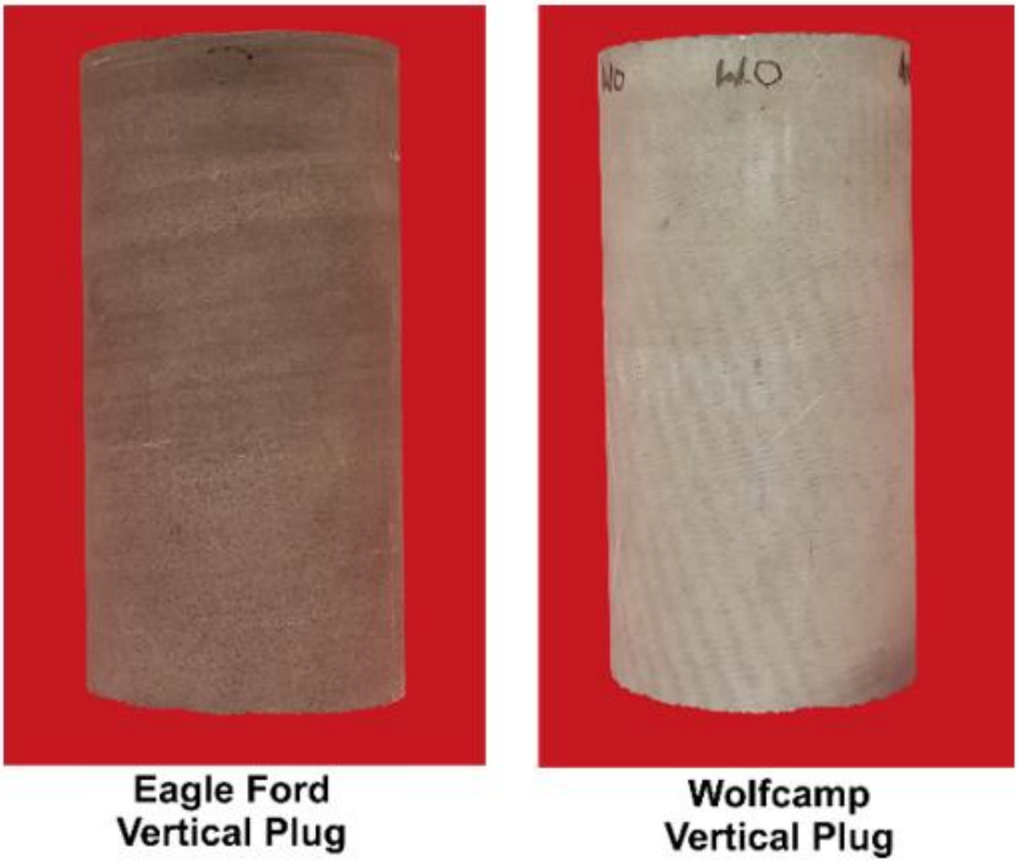

Figure S1. Cylindrically shaped vertical plugs of Eagle Ford and Wolfcamp shales used in construction of disc-shaped samples for Brazilian Indirect Tensile Strength testing.

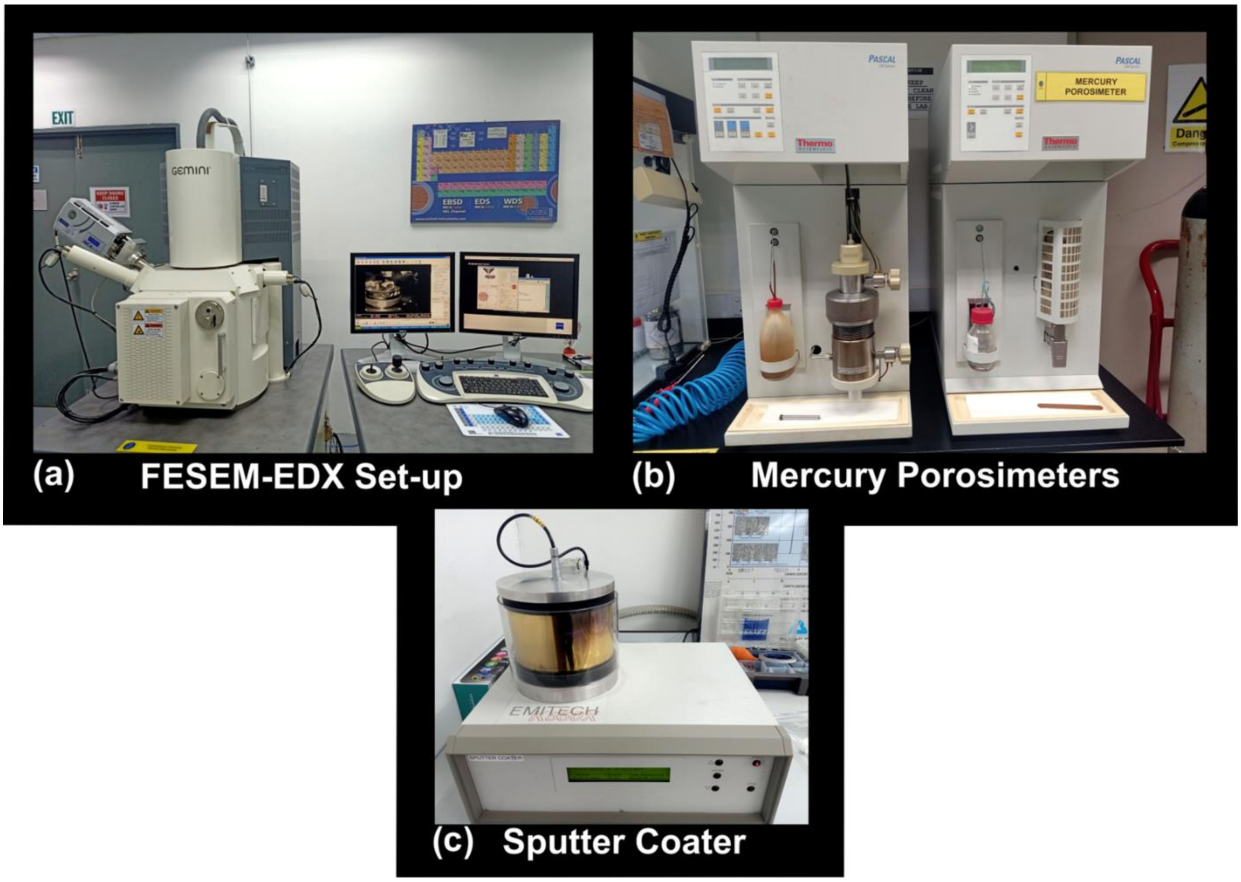

Figure S2. (**a**) FESEM-EDX set-up for mineralogical characterization and elemental composition analysis, (**b**) 140 & 240 Series Mercury Porosimeters for porosity-permeability analysis, and (**c**) Sputter coater for metallic coating of sample splits to aid conductive layering.

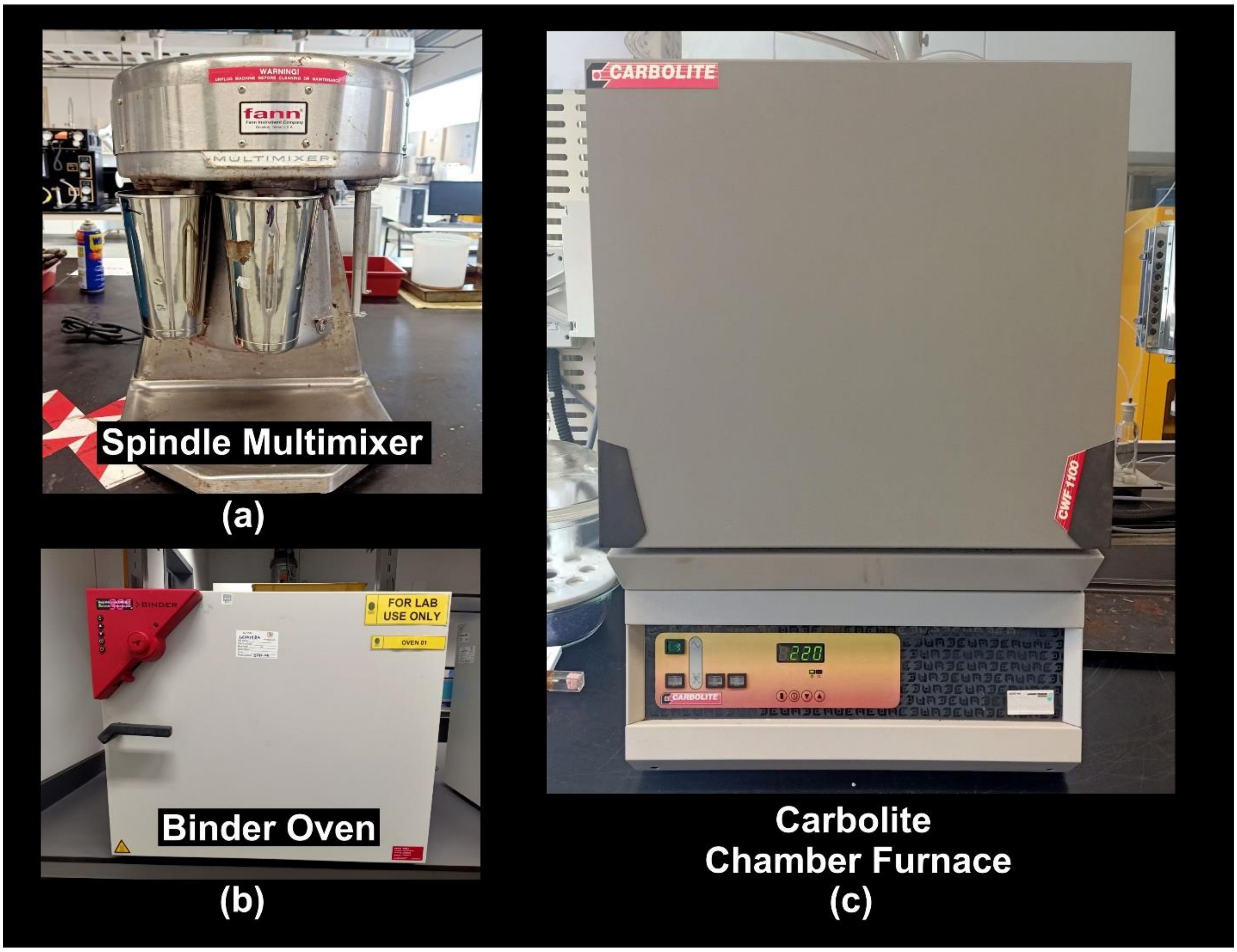

Figure S3. Experimental set-up of (**a**) Model 9B 5-Spindle Multimixer to facilitate homogeneous dissolution of chemical additives, (**b**) Binder oven for thermal conditioning of samples at 90 °C, and (**c**) Carbolite chamber furnace for thermal conditioning of samples at 220 °C .

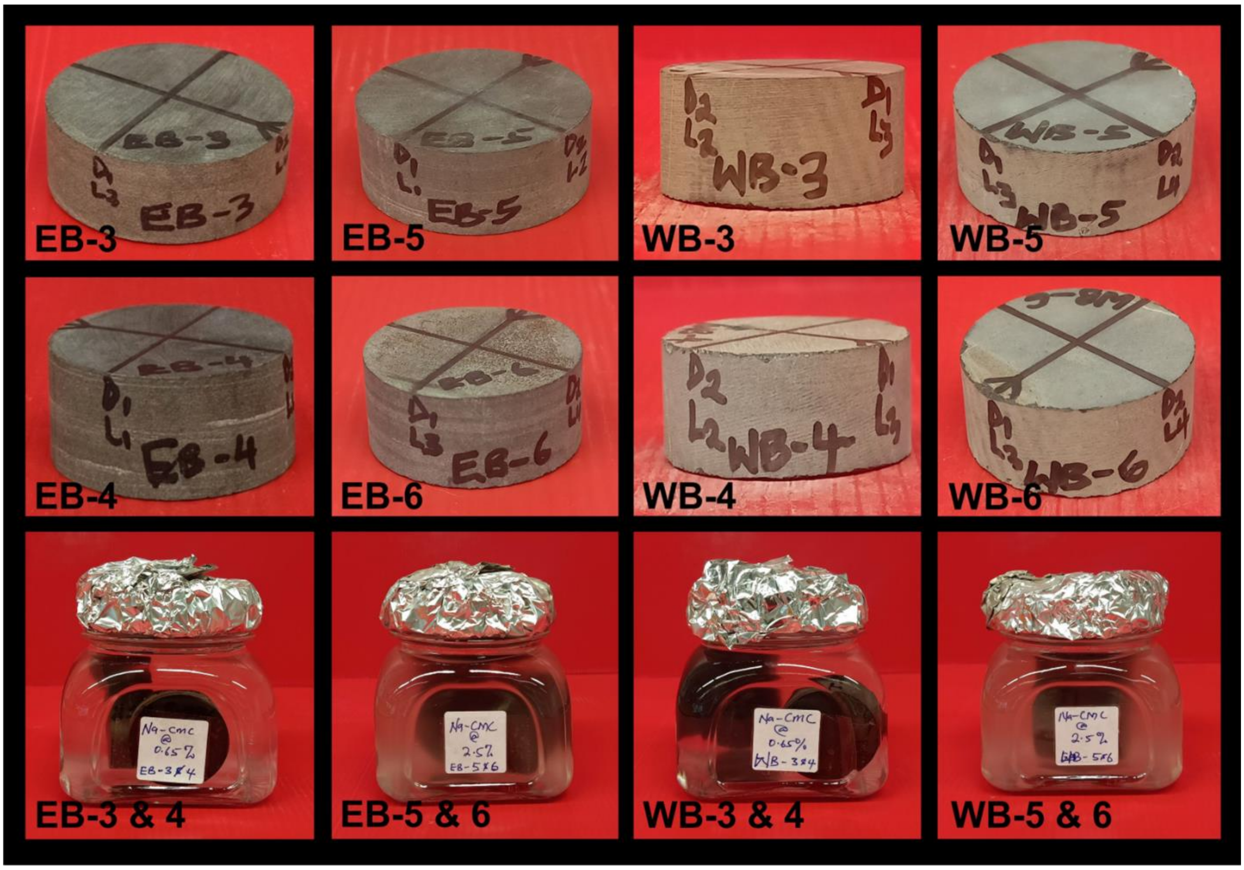

Figure S4. Brazilian disc-shaped samples of Eagle Ford and Wolfcamp shales placed uprightly in pairs in borosilicate glass containers and filled with linear fracturing fluids of polymer concentrations at 0.65 and 2.50% in preparation for thermal conditioning. The borosilicate glass containers were covered with aluminum foils and afterwards were tightly sealed with metallic lids before placement for thermal conditioning.

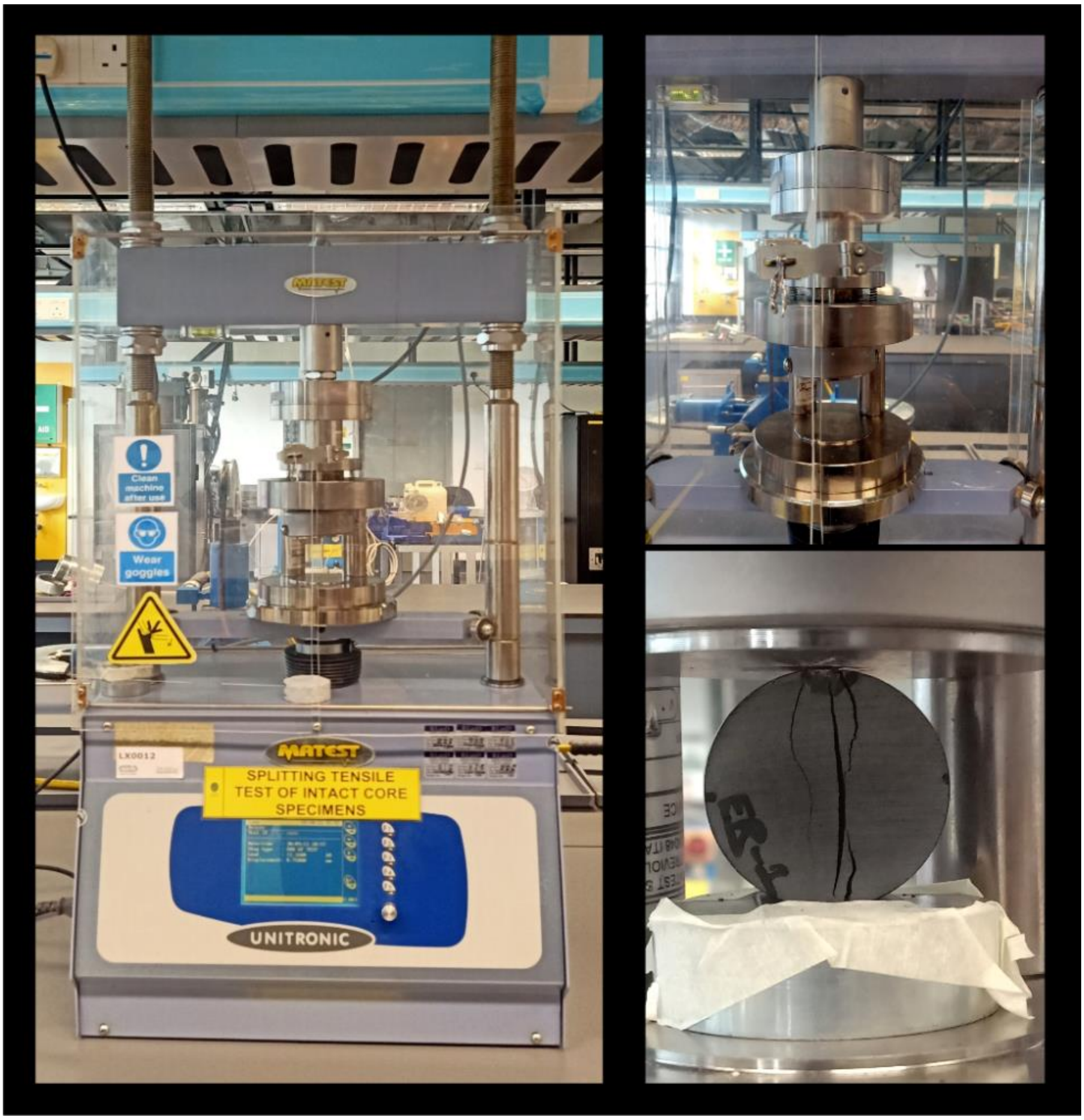

Figure S5. Brazilian indirect tensile strength testing equipment set up with disc-shaped sample of Eagle Ford shale held between loading platens with diameter-concentrated compressional loading applied to failure.
